# Misnomers in Dermatology and Other Medical Specialties—How to Deal with Science Fiction?

**DOI:** 10.3390/jcm15103608

**Published:** 2026-05-08

**Authors:** Vera L. Baumann Griss, Radina V. Velikova, Peter Itin, Deborah R. Vogt, Adam Daunton, Zita-Rose Manjaly Thomas, Michael Geiges, Simon M. Mueller

**Affiliations:** 1 Department of Dermatology, University Hospital Basel, 4031 Basel, Switzerlandsimon.mueller@usb.ch (S.M.M.); 2 Department of Clinical Research, University Hospital Basel, University of Basel, 4001 Basel, Switzerland; 3 Department of Dermatology, Salford Royal NHS Foundation Trust, Salford M6 8HD, UK; 4 Department of Internal Medicine, University Hospital Basel, 4001 Basel, Switzerland; 5 Department of Dermatology, University Hospital Zurich, 8091 Zürich, Switzerland

**Keywords:** misnomer, terminology, dermatological nomenclature, medical errors, mistakes

## Abstract

**Background/Objectives**: A “misnomer” is a term that is erroneous or inaccurate. Dermatology is replete with well-established misnomers such as “Mycosis fungoides”, “keratoacanthoma”, “actinomycosis”, “granuloma pyogenicum”, or “Kaposi sarcoma”. Misnomers have originated from misconceptions or mistranslations that were passed down historically, and reflect the comparative–descriptive origin of terminology. We aimed to collect and categorize (non-)dermatological misnomers and to assess strategies for abandoning them. **Methods**: A questionnaire-based survey with 411 senior academic physicians of five university hospitals in Switzerland was conducted to evaluate the frequency of misnomers across dermatology and other specialties. In addition, a systematic review was conducted, complemented by manual searches to collect medical misnomers. **Results**: The survey and systematic review yielded 536 non-dermatological and 168 dermatological misnomers, pointing to a particular abundance of misnomers in dermatology, which could be categorized into seven categories. A wrong pathological concept was the most common origin of misnomers. Amongst dermatologists, 60.0% regarded misnomers as a relevant problem in their specialty compared to only 35.5% of non-dermatologists. Preferred strategies for abandoning misnomers were avoidance, consensus definitions, and information in teaching. **Conclusions**: Our results show that misnomers are particularly abundant in dermatology, indicating the need to modernize terminology.

## 1. Introduction

A misnomer is a name (or a term) that is erroneous or inappropriate [1]. The term “misnomer” originates from old Law-French “mesnommer” which is composed of the Old French adverbial prefix “mes” meaning “bad, wrong or improper” and the Latin verb “nominare” which means “to name” [2]. Misnomers are commin in everyday language: for example “strawberries” are not truly berries and “peanuts” are not nuts. Such misnomers are usually harmless. However, the use of misnomers can be hazardous in contexts that directly affect human health and safety such as aviation, warfare, or political negotiations [3,4,5,6]. Likewise, correct terminology is essential in medicine to prevent avoidable mistakes that could lead to adverse patient ourcomes. It can be surmised that most medical misnomers have evolved historically. They are usually well-established and regularly used without any harmful potential, such as “vitamin” D, which is not truly a vitamin [7,8]. Such misnomers may be scientifically incorrect, but likely have no clinical relevance. However, there are also misnomers that possibly promote relevant misconceptions or unfavorable treatment situations. They may perpetuate obsolete concepts of the underlying pathogenesis (e.g., “mycotic aneurysm”) [9], lead to misinterpretations of medical findings (e.g., lupus “anticoagulant”) [10,11], result in incorrect diagnoses (e.g., “ring worm”) [12], cause underestimation of the severity of conditions that are mistakenly considered as benign (“keratoacanthoma”) or overestimation (“metastatic Crohn’s disease”), lead to inappropriate treatment (e.g., “actinomycosis”) [13,14], or result in withholding of indicated treatment (e.g., thrombosis of the “superficial femoral vein”) [15].

It has been stated that medical language can be “as misleading as statistics” [16] and misnomers may be contributing to this.

In this vein, dermatology has been described as being particularly rich in misnomers as a consequence of the origins of academic dermatology, including the nosology of skin diseases, which was based upon the description and comparison of morphology [16]. Of note, the shortcomings of the dermatological nomenclature and classification have already been described more than 100 years ago ([17], pages 18–24). Thus, not surprisingly, the first mention of the word “misnomer” in the context of medical terminology has—to the best of our knowledge—been attributed to a dermatologist, Dr. Moses Scholtz from New York City [18]. The call to modernize the dermatological nomenclature and classification goes even back to Galen of Pergamon (AD 133–200), who developed a topographical system. The first systematic textbook on skin diseases was written by Hieronymus Mercurialis (1530–1606), who adopted Galen’s classification but refined it [19].

Considering the presumed abundance of misnomers in dermatology, it seems likely that other perceptual–comparative specialties, such as pathology, radiology, and nuclear medicine (which, together with dermatology, are termed the “perceptual specialties”) [20,21], also contain a relatively high proportion of misnomers compared to “non-perceptual” specialties. However, so far, there are no published data available to corroborate this assumption.

Moreover, it is unknown how many misnomers exist in the medical language, how they are distributed over the specialties, whether clinicians and researchers recognize them as being incorrect, and if they consider them a relevant issue.

The goals of this study were therefore:

1. To estimate the approximate number of misnomers in dermatology;
2. To assess whether perceptual specialties, and in particular dermatology, harbor more misnomers compared to non-perceptual specialties;
3. To assess whether physicians regard misnomers as clinically and/or scientifically relevant;
4. To assess if and how academic physicians intend to abandon misnomers;
5. To introduce so far unreported misnomers collected in the questionnaire-based survey;
6. To categorize the misnomers.

To address the study goals 1–6, a web-based survey targeting at all academic physicians affiliated with the five main university hospitals in Switzerland was conducted. In addition, study goals 1, 2, 5, and 6, respectively, were addressed through a systematic review to collect all publications in the English language containing the term “misnomer” in their title.

## 2. Materials and Methods

### 2.1. Questionnaire

All senior academic physicians of all specialties (“senior” = consultants or more advanced) of the five Swiss University Hospitals (Basel, Bern, Zurich, Lausanne, Geneva) were invited to participate in an anonymous web-based survey.

Participants were asked to estimate the number of misnomers in their own specialty (but not in other specialties), to give examples, as well as suggestions for replacement. Next, participants were asked to rate the relevance of four possible reasons for the development of misnomers (“Professional use of lay terms”, “Misleading word constructions (neologisms)”, “Mistranslations”, “Wrong pathogenic concept”) on a Visual Analogue Scale (VAS 0–10; 0: “not relevant”, 10: “most relevant”). This categorization of misnomers was based on a preliminary review of multiple previous reviews on the underlying reasons for misnomers, listed in Table 1, particularly Subramanyam et al., Hulmani et al., and Barankin and Freiman [7,22,23]. Participants could further provide free text responses outlining any other reasons they considered important for the development and continued use of misnomers. The free-text responses were reviewed and qualitatively analyzed to identify additional themes; however, no substantially new aspects emerged apart from the predefined categories. The relevance of misnomers was assessed as a binary variable (“Are misnomers clinically and/or scientifically relevant?”). Finally, participants were asked to rate the usefulness of three possible strategies for abandoning misnomers (“Avoidance in clinical routine and research” or “Clarification in teaching”, “Development of consensus definitions”) on a VAS (0–10; 0: “not useful”, 10: “most useful”). Participants could further provide free text responses outlining any other strategy they considered helpful for abandoning misnomers.

### 2.2. Statistical Analysis

A total of 651 participants answered at least one question of the online questionnaire. The full analysis set (FAS) includes 411 participants who answered at least one question other than demographic characteristics.

The (estimated) number of misnomers was analyzed for an association with gender, age, language region (French vs. German-speaking parts of Switzerland), and medical specialty. The various medical specialties, other than dermatology, were combined into four broad categories: perceptual specialties, surgical specialties, medical specialties, and “other” specialties (Supplementary Text S1). Dermatology was set as the reference level for model contrasts. Due to extreme zero-inflation (163/410 participants indicated that there are no misnomers in their discipline), we used a two-part model (hurdle model). Relevance of misnomers (binary) was analyzed using a mixed-effects logistic regression model. Relevance of reasons for the origin of misnomers and usefulness of strategies to abandon misnomers (VAS) were analyzed visually by means of radar charts (Supplementary Text S2). All statistical analyses were performed by biostatisticians using the software R, version 3.2.1.

### 2.3. Systematic Review

The databases PubMed (search term “misnomer [Ti]”), Embase (filter setting AND “title”), Web of Science (filter setting AND “title), Scopus (filter setting AND “title), and Cochrane (filter setting AND “title) were searched in January 2017 for publications including the term misnomer in the title. Non-English publications, duplicates, non-medical, and veterinarian misnomers were removed from further analysis (the excluded German and French misnomers are listed in Supplementary Texts S3 and S4). Thereafter, all search hits were evaluated for dermatological content to be labeled as “dermatological” and/or “non-dermatological” (see the flow chart in Supplementary Text S2). In addition, in reviews on misnomers (*n* = 7, [7,18,22,23,24,25,26]), hand searching of the reference lists (snowballing) was performed, and relevant publications were evaluated accordingly.

## 3. Results

### 3.1. Number of Misnomers in Dermatology

The search of the databases and the survey (410 answers) yielded 168 dermatological misnomers (Table 1 and Supplementary Text S5).

#### 3.1.1. Number of Dermatological and Non-Dermatological Misnomers According to the Searched Databases

The searches for misnomers yielded 507 medical misnomers, of which 168 (32.8%) were dermatology-related (Table 1, Supplementary Texts S6 and S7), indicating a disproportionate abundance of misnomers in dermatology.

#### 3.1.2. Number of Dermatological and Non-Dermatological Misnomers According to the Online Survey

**Figure 1:**
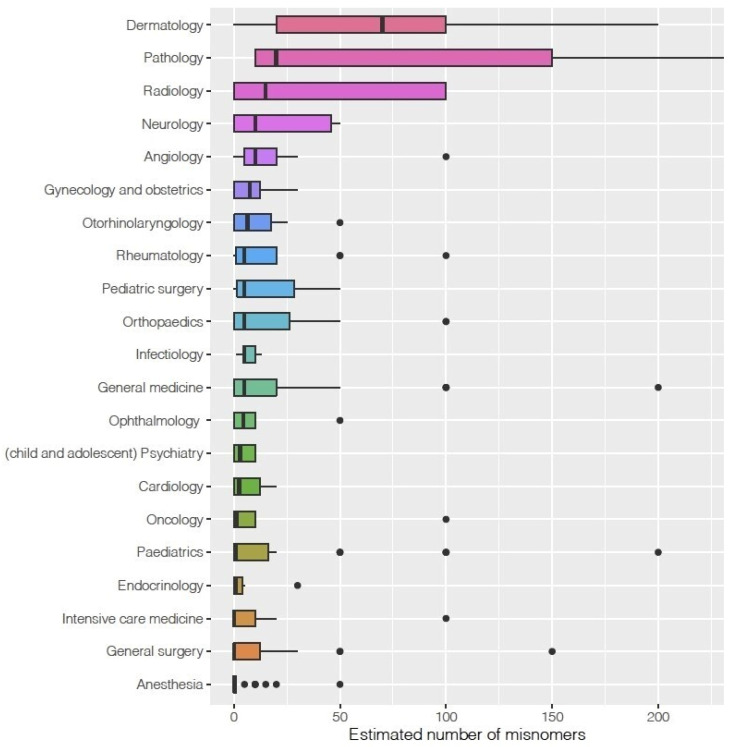
Number of misnomers (0–220) per medical specialty (as estimated by the participants). The X-axis is cut at 220 for better visualization; 13 data points (outliers) were excluded from the figure for better visualization. Only specialties with >7 participants are listed.

In total, 3285 invitations to the survey were sent, 651 participants started the survey, and 411 (response rate 12.5%) answered at least one question in addition to demographics (median age was 45 years (28–76 years), 282 (68.8%) men). A total of 41 different medical specialties participated (Figure 1).

Misnomers were indicated across all medical specialties, resulting in a total of 65 dermatological and 211 non-dermatological misnomers (Supplementary Texts S8 and S9). Of the latter, 19 have not yet been published (Supplementary Text S10); selected examples are presented in Table 2.

### 3.2. Presence and Estimated Numbers of Misnomers Analyzed by Gender, Age, Language Region, and Specialty

The median estimated number of misnomers per medical specialty ranged from 0 (anesthesia, intensive care medicine, general surgery) to 70 (dermatology; Figure 1). Dermatologists were more likely to confirm the use of misnomers in their specialty (Table 3) and estimated these to be more frequent (Table 4) than participants from other specialties. The difference was smallest compared to the other perceptual specialties. German-speaking participants were more likely to confirm the use of misnomers in their specialty and estimated the number of these to be higher compared to French-speaking participants. Older participants estimated the number of misnomers lower (Table 4) than younger participants. We found no indications for an association with gender (Table 3 and Table 4).

### 3.3. Assessment of the Clinical and Scientific Relevance of Misnomers

Overall, 35.0% of participants considered misnomers to be a relevant problem. The proportion of dermatologists (60.0%) with this perception was clearly higher compared to other specialties (41.0%; odds ratio OR [95% confidence interval]: 0.41 [0.16, 1.02], *p*-value = 0.056), vs. medical specialties (33.0%; OR 0.30 [0.12, 0.73], *p* = 0.008), vs. surgical specialties (30.0%; OR 0.26 [0.10, 0.66], *p* = 0.005), and vs. perceptual specialties (22.0%; OR 0.17 [0.04, 0.69] *p* = 0.013). We found no evidence for an association with gender (females vs. males: OR 1.18 [0.73, 1.92], *p* = 0.50), age (10-year increase: OR 0.94 [0.72, 1.23], *p* = 0.67), or language (French vs. German: 0.65 [0.36, 1.18], *p* = 0.16).

**Figure 2:**
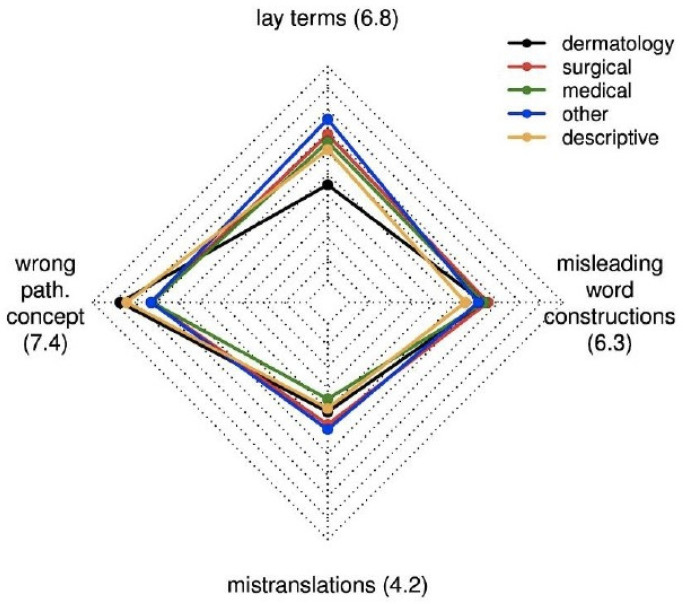
Radar chart for the median estimated importance of lay terms, wrong pathogenic concepts, mistranslations, and misleading word constructs for the development of misnomers for each medical category. Each axis represents one item, with values on a Visual Analogue Scale (VAS) from 0 to 10, increasing from the center outward.

The participants considered a wrong pathological concept the most relevant reason for the development of misnomers (median [interquartile range] VAS: 7.4 [6.0, 8.5]) and mistranslations the least relevant (4.2 [2.5, 6.5]; Figure 2). Ratings of participants from other perceptual specialties were closest to those of dermatologists.

Further reasons for misnomers mentioned by the participants were adoption of brand names or drugs for a medical procedure or term (e.g., “warfarinise”), abbreviations (e.g., “CVI” for “cerebrovascular insufficiency/insult”, “chronic venous insufficiency”, “common variable immunodeficiency”), or eponyms such as “Paget’s disease of the breast” or “Erythroplasia de Queyrat”.

### 3.4. Strategies for Abandoning Misnomers as Suggested by the Participants

**Figure 3:**
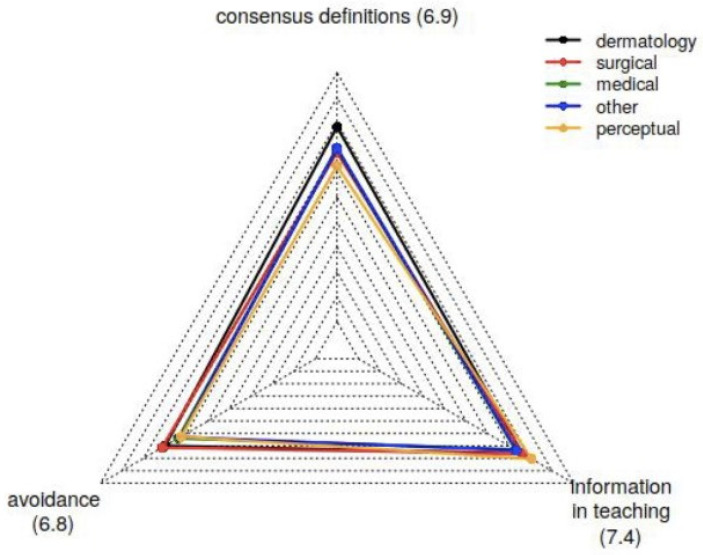
Radar chart displaying the median estimated impact of consensus definition, avoidance in clinical daily routine, and information in teaching as options to eliminate misnomers for each category of medical specialty. Each axis represents one item, with values on a Visual Analogue Scale (VAS) from 0 to 10, increasing from the center outward.

Participants rated the importance of the three provided options for abandoning misnomers (“Avoidance in clinical routine and research”, “Clarification in teaching”, and “Development of consensus definitions”) more or less equally (Figure 3). Consensus definitions were considered most important by dermatologists.

Other suggestions mentioned most often were “agreement to one term only per disease” and “avoidance of misnomers in public media”.

### 3.5. Categorization of Medical Misnomers

Once the misnomers were collected, we attempted to categorize them according to the strategies mentioned above into seven themes (Table 5 and Supplementary Text S5).

## 4. Discussion

Medical language has evolved from Greek and Latin through national languages to today’s English-dominated scientific discourse, leaving numerous historical traces in terminology. While some terms remain valid, others are outdated or misleading. Certain misnomers persist for historical reasons and remain in use despite advances in medical knowledge and technology that have clarified the underlying disease mechanisms (e.g., “*Mycosis fungoides*” or “*Kaposi sarcoma*”). Despite the era of evidence-based precision medicine, misnomers are not systematically addressed in major standardization initiatives such as SNOMED CT (Systematized Nomenclature of Medicine—Clinical Terms) [27] or the classification of diseases like the ICD-11 (International Classification of Diseases, 11th Revision) [28]. Nor are they explicitly considered in medical education, error research, or quality improvement. Consequently, awareness of medical misnomers and their implications remains limited. This project provides a clinical and database-based overview of medical misnomers, with a particular focus on dermatology.

### 4.1. Identified Misnomers, Their Categorization, and Their High Abundance in Dermatology and Other Perceptual Specialties

In our questionnaire-based study of 411 senior academic physicians, complemented by a systematic review, more than one quarter (168/536) of all identified misnomers were dermatology-related, highlighting that this specialty is disproportionately rich in misnomers. Presumably, even more misnomers exist in dermatology and other specialties, considering that we identified 19 that had not previously been described in any survey. Interestingly, other perceptual specialties (“perceptual” according to Johnson and Berner [20,21]) also showed higher numbers of misnomers (see Figure 1) compared with non-perceptual specialties. This finding supports our hypothesis that misnomers are particularly abundant in these fields, possibly due to the descriptive–comparative origins of their nomenclature. The leading position of dermatology with regard to misnomers may also be attributable to the large number—approximately 4000—of distinct diagnoses within the specialty [29].

The collected misnomers were initially categorized into four themes: “Wrong pathogenetic concepts”, “Professional use of lay terms,” “Neologisms/Misleading word constructions,” and “Mistranslations”. The survey responses suggested adding “Abbreviations”—which may cause dosage or diagnostic errors—and “Eponyms,” which can perpetuate misnomers and misconceptions (e.g., “*Erythroplasia de Queyrat*”). We further propose a seventh category, “Analogies and resemblances,” for terms such as “*buffalo hump*”, “*mechanic’s hands*”, “*raccoon eyes*”, “*pepper spot skull*”, or “*sausage digits*”. While future research may refine this categorization, the seven categories provide a practical framework for sorting medical misnomers.

### 4.2. The Relevance of Misnomers

Inaccurate concepts are a key reason why misnomers matter. They range from seemingly “innocent” analogies to clearly misleading terminology. For example, thrombosis of the “*arteria femoralis superficialis*” requires anticoagulation because it is actually a “deep” vein. Likewise, MRSA is not only resistant to methicillin; “*actinomycosis*” is not a fungal infection, and “*cryptococcosis*” is not bacterial [29]. Misnomers can also have serious clinical consequences: the term “*sporadic cretinism*” may have delayed adequate treatment in children, resulting in preventable mental retardation, as it was confused with “true” or “*epidemic cretinism,*” in which mental impairment is acquired in utero [13].

Misconceptions resulting from misnomers can, of course, also occur in dermatology. For instance, a patient with mycosis fungoides was recently referred to our department after ineffective treatment with systemic fluconazole, prescribed by non-dermatologists for this diagnosis. While this case may be exceptional, more common challenges arise from dermatological nomenclature, particularly in the terminology of melanocytic lesions. The term “*melanoma*” is itself a misnomer, as the suffix “-oma” typically implies a benign tumor. Atypical melanocytic and “spitzoid” lesions (e.g., “*Spitz nevi*”, “*atypical Spitz tumors*”, and “*spitzoid melanomas*”) are often histologically challenging, with high inter-observer variability [30,31]. Their correct terminology is particularly important as the label given to a lesion is decision-making for management, which can vary from local excision only to wide re-excision, sentinel lymph node biopsy, long-term follow-up [32], or even melanoma-specific treatment. Another critical term is “*dysplastic naevus*,” which has repeatedly been designated a misnomer [33,34] partly because the underlying concept is flawed and partly because it may lead to both over- and under-treatment of these lesions [35]. Irrespective of such academic semantics, many clinicians consider misnomers clinically relevant: in our survey, one in five to one in three non-dermatologist participants regarded them as important, and nearly two-thirds of dermatologists deemed them clinically significant, suggesting that misnomers may have particularly important implications in dermatology.

### 4.3. How to Abandon Misnomers?

The participants rated the impact of the three options—“Avoidance”, “Information in teaching”, and “Consensus definitions”- as almost equally important. Based on these results, a four-step approach could be considered:

1. Raise awareness: Increase recognition of misnomers and/or critical terminology in scientific publications, at conferences, and through teaching to sensitize clinicians, educators, and researchers to their potential impact.
2. Systematic collection and evaluation: Experts and boards of medical specialties could compile misnomers in structured databases for evaluation, with open-access platforms and blog sites (e.g., http://medicalmisnomers.blogspot.com/, accessed on 26 April 2026) serving as additional resources for discussion.
3. Marking and standardization: Misnomers should be clearly marked—for example, with quotation marks, italics, or the annotation “(mn)” in the medical literature (e.g., *“mycosis fungoides” (mn)*)—and critically reviewed in expert-led consensus definitions reflecting current scientific knowledge [36,37,38,39,40,41,42].
4. Replacement in a standardized manner: Misnomers could be replaced when appropriate. As the legendary Canadian physician Sir William Osler (1849–1919)—to whom several eponyms are attributed, including Osler’s nodes, Osler–Weber–Rendu disease, and Osler’s sign—advised in 1902, “Use guidelines for naming diseases. If our knowledge does not permit giving a name pointing to the aetiology of the disease, the rule should be to pick the one which seems least objectionable” [43].

### 4.4. Recent Advances in Dermatologic Nomenclature and Outlook

More recent publications highlight the need to refine classification systems. For example, a recent report questioned whether “*desmoplastic melanocytic naevus*” represents an overlooked diagnosis or whether the label itself may be misleading, underscoring how emerging entities can expose weaknesses in established terminology [44]. Likewise, the long-standing term ILVEN (Inflammatory Linear Verrucous Epidermal Nevus) has been challenged by the proposal of a more accurate nomenclature—MALID (Mosaicism Associated Linear Inflammatory Dermatosis), exemplifying how genotype–phenotype insights can drive renaming efforts [45]. Even contemporary practice terminology may generate new “misnomers”, as argued for the term “*sunscreen doping*”, which has been used to describe the addition of unapproved UV-absorbing substances to sunscreen formulations under the label of non-active ingredients. This illustrates that misnaming is not limited to classical disease entities but can extend to behavioral or cosmetic-dermatology concepts [46]. Modern technologies, including targeted and multi-omics approaches that explore gene expression patterns at the protein and RNA levels, allow the identification of historical misnomers such as “*Merkel cell carcinoma*”, which is unlikely to be derived from Merkel cells and may instead originate from multiple or divergent cell types, including those of B-cell lineage [47]. In addition, recent discussions have highlighted potentially misleading or inconsistently used terms in routine dermatologic communication, including the distinction between chilblains (als referred to as perniones) and chilblain lupus (more precisely framed as idiopathic perniosis versus lupus-associated perniosis), as well as eosinophilic fasciitis (often referred to as Shulman syndrome), nevus simplex versus nevus flammeus (better communicated as salmon patch versus port-wine birthmark), and hand, foot, and mouth disease (sometimes expanded to hand–foot–mouth and buttocks disease)- each illustrating how terminology can blur etiologic meaning, clinical boundaries, or patient expectations [48]. Additional misnomers in dermatology that were neither captured in our systematic review nor identified in the questionnaire-based part may arise from dermoscopic terminology, where many descriptors are based on resemblance to familiar visual analogies independent of the underlying pathology (e.g., “*hairpin vessels*”, “*starburst pattern*” (resembling a stellar explosion), “*leaf-like structures*”, “*spoke-wheel structures*”, “*blue-white veil*”). The frequent use of suffixes such as “-like” may partly mitigate the potential misnomer character of these terms. For instance, the so-called “*rainbow pattern*”, described in Kaposi sarcoma, does not represent a true spectrum of colors but rather an optical phenomenon observed under polarized light [49]. In addition, “*pseudopods*” is a misnomer because the term, originally used in biology for dynamic cellular extensions, refers in dermatoscopy to static, tissue-level pigment projections rather than motile structures.

In parallel, structured nomenclature initiatives- such as the recently proposed updated classification frameworks for EDDs (Epidermal Differentiation Disorders)- demonstrate how consensus-driven reclassification can reduce ambiguity and improve clinical communication [50,51]. Notably, incorporating gene-based naming (e.g., using a gene label for syndromic EDDs with prominent extracutaneous involvement, as in a disease associated with PHYH (phytanoyl-CoA hydroxylase), formerly termed Refsum syndrome) may facilitate recognition by specialists outside dermatology [50]. Conversely, when skin involvement is not the dominant feature, the umbrella “EDD” terminology can be complemented or replaced by descriptors reflecting the primary organ system affected [50].

Medical language is evolving slowly but steadily, and new misnomers will likely continue to appear. The advent of “-omics” and personalized medicine, driven by integration with disciplines such as computational biology and bioinformatics [46], brings both challenges and opportunities. These influences provide a chance to establish a more structured nomenclature that is less prone to misnomers. A notable example is the systematic naming of therapeutic monoclonal antibodies by the World Health Organization using the International Nonproprietary Name method, which encodes target and species information [47]. Names are created by assembling an individual prefix, a target substem (e.g., “-li” for immunomodulating), and the universal stem “-mab” for monoclonal antibodies [47,48]. The introduction of the ICD-11 classification, and in particular the ICDD (ICD-11 Classification of Dermatological Diseases), shows how medical terminology and classification systems have evolved over time and now contribute to reducing ambiguity and improving clinical communication. By providing a structured and consensus-based framework, the ICDD has the potential to bring greater order to the historically heterogeneous dermatological nomenclature [52].

Beyond structured classification systems such as the ICDD, the current disease ontology itself has also been questioned. The Translational Dermatology Initiative points out that traditional classifications of inflammatory skin diseases do not adequately reflect molecular heterogeneity and the dynamic nature of these conditions. By proposing a molecularly driven disease classification that is independent of conventional diagnostic labels, this initiative represents another important step toward greater conceptual clarity and precision in dermatology [53].

### 4.5. Limitations

The data from our survey is limited by a response rate of 12.5%. However, this response rate is within the expected range of non-remunerated online surveys. Importantly, selection bias cannot be excluded, as physicians with a particular interest in terminology and nomenclature may have been more likely to participate, potentially leading to an overrepresentation of this perspective. Generalizability to other countries may be limited by national factors such as appreciation for historically used (dermatological) terms, knowledge of dermatological diseases in non-dermatologists, and language. In addition, differences between languages (e.g., German, French, and English) may influence how certain terms are understood and used, which could affect the identification and classification of misnomers. The literature search was limited to publications containing the term “misnomer,” which may have excluded relevant studies using alternative terminology (e.g., “misleading term” or “outdated nomenclature”). Furthermore, although the questionnaire was internally reviewed and pilot-tested, no formal psychometric validation was performed, which may limit the robustness of the survey instrument.

## 5. Conclusions

Medical misnomers are common in many specialties, but appear to be particularly abundant in dermatology. The main reasons for their persistence include outdated pathogenic concepts, misleading word constructions, mistranslations, and descriptive analogies that have been retained in the literature over time. Our findings from both the literature review and the questionnaire conducted among senior academic physicians suggest that these terms are not merely linguistic curiosities but may impact understanding and communication in clinical practice. In recent years, however, changes have been introduced both in dermatology and in international disease classification systems, aiming to bring greater clarity and precision to medical language. Like medicine itself, medical terminology continues to evolve. Although this process is often slow, it reflects an ongoing effort to refine dermatological terminology and improve interdisciplinary understanding.

## Figures and Tables

**Table 1:** Summary of the database searches for misnomers divided into “dermatological” versus “non-dermatological” misnomers.

	Number of Hits for “Misnomer”	Dermatological Misnomers; *n* (Percentage)	Non-Dermatological Misnomers; *n* (Percentage)
Databases			
PubMed	347	36 (12.77)	246 (87.23)
Embase	373	22 (8.46)	238 (91.54)
Web of Science	543	22 (8.63)	233 (91.37)
Scopus	531	31 (11.36)	242 (88.64)
Cochrane	1	0 (0)	1 (100)
Reviews on misnomers			
Hulmani [22]	86	86	0
Barankin [23]	47	47	0
Nosrati [24]	16	16	0
Savitha [25]	80	80	0
Subramanyam [7]	36	16	0
Scholtz [18]	25	25	0
Danesh [26]	4	4	0
Total (multiple mentions counted only once)	507	168 (32.81)	339 (67.18)

**Table 2:** Selected examples of unreported misnomers collected in the questionnaire-based survey, including explanations explanations of why they are considered misnomers and suggestions for improved terminology provided by the participants.

Misnomer	Explanation	Suggestion for Improvement
1. Basalioma	Ending suggests a benign disease	Basal cell carcinoma
2. Congenital nevus	Is neither hereditary nor present at birth	Infantile nevus
3. Eosinophilic cellulitis	Not an infection of the subcutis but the dermis	Eosinophilic dermatitis
4. Erythema e pudore	Not only triggered by shame, but also by any involuntary flushing associated with emotions	Emotional erythema
5. Granuloma telangiectaticum	Not granulomatous	Reactive vascular proliferation syndrome, telangiectatic type
6. Lentigo maligna	Lentigo suggests a benign disease	Melanoma in situ
7. Lichen ruber/planus	Purely descriptive, does not capture pathophysiology; moreover, “ruber” and “planus” may not be appropriate as other colors and elevated/hyperkeratotic forms exist	No suggestion made
8. Lichen sclerosus	Purely descriptive, does not capture pathophysiology	No suggestion made
9. Malignant melanoma	The suffix “-oma” suggests benignity. The addition of the adjective “malign” to melanoma (common in German-speaking areas) is also misleading because it suggests the existence of a “benign” melanoma	Melanoma, or more precisely, melanocytic malignancy
10. Mallorca acne	Does not occur only in Mallorca	Suncream/bath oil acne
11. Mycid	Does not reflect the clinical presentation	Hyperergic reaction to dermatophytes
12. Neurodermatitis/Neurodermitis	Not a primary disease of skin nerves	Atopic dermatitis
13. Pemphigus chronicus benignus familiaris Hailey-Hailey	Eponym, moreover, it is not pemphigus/an autoimmune bullous disease	Acantholytic dermatitis due to ATP2C1 gene mutation
14. Pityriasis lichenoides acuta/chronica	Purely descriptive, does not reflect the underlying pathophysiology	No suggestion made

**Table 3:** Estimates from the binomial mixed-effects model predicting the presence of misnomers.

	Estimate	95% CI	z	* p * (>|z|)
Intercept	13.753	[3.175, 59.908]	3.49	<0.001
Gender (female versus male)	0.876	[0.559,1.371]	−0.58	0.562
Age (10-year increase)	0.999	[0.776,1.285]	−0.01	0.991
Language region (French versus German)	0.574	[0.346,0.951]	−2.16	0.031
Surgical specialties versus dermatology	0.120	[0.027,0.540]	−2.76	0.006
Medical specialties versus dermatology	0.169	[0.038,0.755]	−2.33	0.020
Other specialties versus dermatology	0.073	[0.016,0.327]	−3.42	<0.001
Other perceptual specialties versus dermatology	0.184	[0.033,1.017]	−1.94	0.052

The model predicts the presence of misnomers (zeros vs. non-zeros) by gender, age, language region, specialty, and participants. The estimate for the intercept is odds and odds ratio (OR) for all other variables. A hospital within the language region was included as a nested random effect with a random intercept. The column “95% CI” indicates the limits of the 95% confidence intervals for the OR. A significant difference is indicated if OR is different from 1.

**Table 4:** Estimates from the truncated negative binomial mixed effects model predicting the estimated number of misnomers.

	Estimate	95% CI	z	* p * (>|z|)
Intercept	198.412	[74.623,527.553]	10.60	<0.001
Gender (female versus male)	0.771	[0.437,1.362]	−0.90	0.370
Age (10-year increase)	0.733	[0.546,0.984]	−2.07	0.039
Language region (French versus German)	0.288	[0.125,0.666]	−2.91	0.004
Surgical specialties versus dermatology	0.083	[0.030,0.231]	−4.76	<0.001
Medical specialties versus dermatology	0.192	[0.072,0.513]	−3.29	0.001
Other specialties versus dermatology	0.322	[0.0104,0.998]	−1.96	0.050
Other perceptual specialties versus dermatology	0.549	[0.145,2.081]	−0.88	0.378

The model predicts the estimated number of misnomers (non-zeros) by gender, age, language region, and specialty of participants. Hospital-based workplace within the language region was included as a nested random effect with a random intercept. The column “95% Cl” indicates the limits of the 95% confidence intervals for the estimate. A significant difference is indicated if the limits do not include 1.

**Table 5:** Proposed categorization of medical misnomers.

Type of Misnomer	Examples
Wrong pathogenic concepts	Glioblastoma multiforme, botryomycosis, incontinentia pigmenti, actinomycosis
Professional use of lay terms	Stroke, cancer, hay fever, shingles, hip fracture, skin tags, color blindness, whiplash injury
Analogies and resemblances	Raccoon eyes, butterfly rash, dirty neck, tripe palms, strawberry gingiva, nutmeg liver, port-wine stain
Eponyms	Erythroplasia de Queyrat, fibroepithelioma Pinkus, pityriasis rosea Gibert, Lyme’s disease
Neologisms/Misleading word constructions	Carcinoid, calciphylaxis, antibiosis, gestosis, warfarinise
Abbreviations	“IU” for “international units” insulin: may be mistaken for “10” or “IV” (intravenously)
Mistranslations	Chicken pox (from “chiche pois”), grenz radiation (“grenz” was not a person), grenz zone (histopathology), “annular” (instead of anular)

## Data Availability

The data presented in this study are available on request from the corresponding author.

## Supplementary Materials

The following supporting information can be downloaded at https://www.mdpi.com/article/10.3390/jcm15103608/s1, Supplementary Text S1: List of medical specialties; Supplementary Text S2: Detailed Methods; Figure S1: Flow diagram of the database search and study selection; Supplementary Text S3: German misnomers; Supplementary Text S4: French misnomers; Supplementary Text S5: Dermatological misnomers; Supplementary Text S6: Dermatological misnomers from database search; Supplementary Text S7: Non-dermatological misnomers from database search; Supplementary Text S8: Dermatological misnomers from questionnaire; Supplementary Text S9: Non-dermatological misnomers from questionnaire; Supplementary Text S10: Introduction of unreported misnomers; Table S1: Unreported misnomers with explanations and suggested terminology. The Supplementary Materials contain their own reference list for citations appearing in the supplementary document.
