# Supplementary material for: Misnomers in Dermatology and Other Medical Specialties—How to Deal with Science Fiction?
Source: J Clin Med. 2026 May 8;15(10):3608. doi: 10.3390/jcm15103608 (PMC13207726; doi:10.3390/jcm15103608)
Supplement: Supplementary file 1 [file jcm-15-03608-s001.zip › jcm-4248215-supplementary.pdf]

Supplementary materials

Supplementary Text S1: List of medical specialties, other than dermatology, combined into four broad categories

Perceptual disciplines

1. Nuclear medicine
2. Pathology
3. Radiology

Surgical disciplines

4. General surgery
5. Gynaecology and obstetrics
6. Cardiac surgery
7. HNO
8. Paediatric surgery
9. Oral and maxillofacial surgery
10. Neurosurgery
11. Ophthalmology
12. Orthopaedics
13. Plastic reconstructive and aesthetic surgery
14. Thoracic surgery
15. Traumatology
16. Urology

Medical disciplines

17. General medicine
18. Angiology
19. Endocrinology
20. Gastroenterology
21. Haematology
22. Infectiology
23. Intensive care medicine
24. Cardiology
25. Neurology
26. Emergency medicine
27. Oncology
28. Physical medicine and rehabilitation
29. Pneumology
30. Rheumatology
31. Nephrology and transplantation immunology

Other disciplines

32. Anaesthesia
33. Clinical pharmacology and toxicology
34. Laboratory medicine
35. Paediatrics
36. Child and adolescent psychiatry
37. Psychosomatic medicine
38. Radiation oncology
39. Forensic medicine
40. Social and preventive medicine

Supplementary Text S2: Detailed Methods

Questionnaire

All senior academic physicians of all specialties of the five Swiss University Hospitals (Basel, Bern, Zurich, Lausanne, Geneva) were invited in February 2015 to participate in an anonymous web-based survey (n = 651). Participants were approached by e-mail including a link to log into the survey. The questions were available both in French and German. The collected German and French misnomers were translated into English, and evaluated by a native speaker clinician.

We assessed demography (age, sex, medical discipline, language region) and professional status. Participants were asked to estimate the number of misnomers in their own specialty (i.e. not in other specialities), to give examples, as well as suggestions for replacement. Further, participants were asked to rate the relevance of four possible reasons for the development of misnomers (“Professional use of lay terms”, “Misleading word constructions (neologisms)”, “Mistranslations”, “Wrong pathogenic concept”) on a visual analogue scale (VAS 0–10; 0: “not relevant”,10: “most relevant”). This categorisation of misnomers has been made following a preliminary review of multiple previous reviews on the underlying reasons for misnomers [1-3]. Participants could further outline any other reasons they considered important for the development and continued use of misnomers as free text. The relevance of misnomers was assessed as binary variable (“Are misnomers clinically and/or scientifically relevant?”). Finally, participants were asked to rate the usefulness of three possible strategies to abandon misnomers (“avoidance in clinical routine and research”, “clarification in teaching”, “development of consensus definitions”) on a VAS (0–10; 0: “not useful”, 10: “most useful”). Participants could further outline any other strategy they considered helpful to abandon misnomers as free text.

Statistical Analysis

A total of 651 participants answered at least one question of the online questionnaire. The full analysis set (FAS) includes 411 participants who answered at least one question other than demographic characteristics. All statistical analyses were performed on the FAS, using the statistical software R, version 3.2.1. Statistical analyses were pre-planned in a statistical report and analysis plan.

The estimated number of misnomers was analysed for an association with gender (female vs. male), age (10-year increase), language region (French vs German) and medical discipline. The various medical specialties, other than dermatology, were combined in four broad categories: perceptual specialties (nuclear medicine, pathology, radiology), surgical specialties (general surgery, gynecology and obstetrics, cardiac surgery, HNO, pediatric surgery, oral and maxillofacial surgery, neurosurgery, ophthalmology , orthopaedics, plastic reconstructive and aesthetic surgery, thoracic surgery, traumatology, urology), medical specialties (general medicine, angiology, endocrinology, gastroenterology, hematology, infectiology, intensive care medicine, cardiology, neurology, emergency medicine, oncology, physical medicine and rehabilitation, pneumology, rheumatology, nephrology and transplantation immunology), and other specialties (anaesthesia, clinical pharmacology and toxicology, laboratory medicine, paediatrics, child and adolescent psychiatry, psychosomatic medicine, radiation oncology, forensic medicine, social and preventive medicine). Dermatology was set as reference level for model contrasts. Due to extreme zero-inflation (163/410 participants indicated that there are no misnomers in their discipline), we used a two-part model (hurdle model). The first part analysed the outcome as binary variable (> 0 vs 0), using a mixed-effects logistic regression model; the second part analysed all values > 0, using a truncated, negative binomial (zero-adjusted negative binomial – ZANB) mixed effects model. Both models included hospital nested within language region as random effect with random intercept. The two parts of the model were fit separately, using the R packages lme4 and glmmADMB, respectively.

Relevance of misnomers (binary) was analysed using a mixed-effects logistic regression model including the same fixed and random effects as described above.

Relevance of reasons for the origin of misnomers and usefulness of strategies to abandon misnomers (VAS) were analysed visually by means of radar charts.

Systematic Review

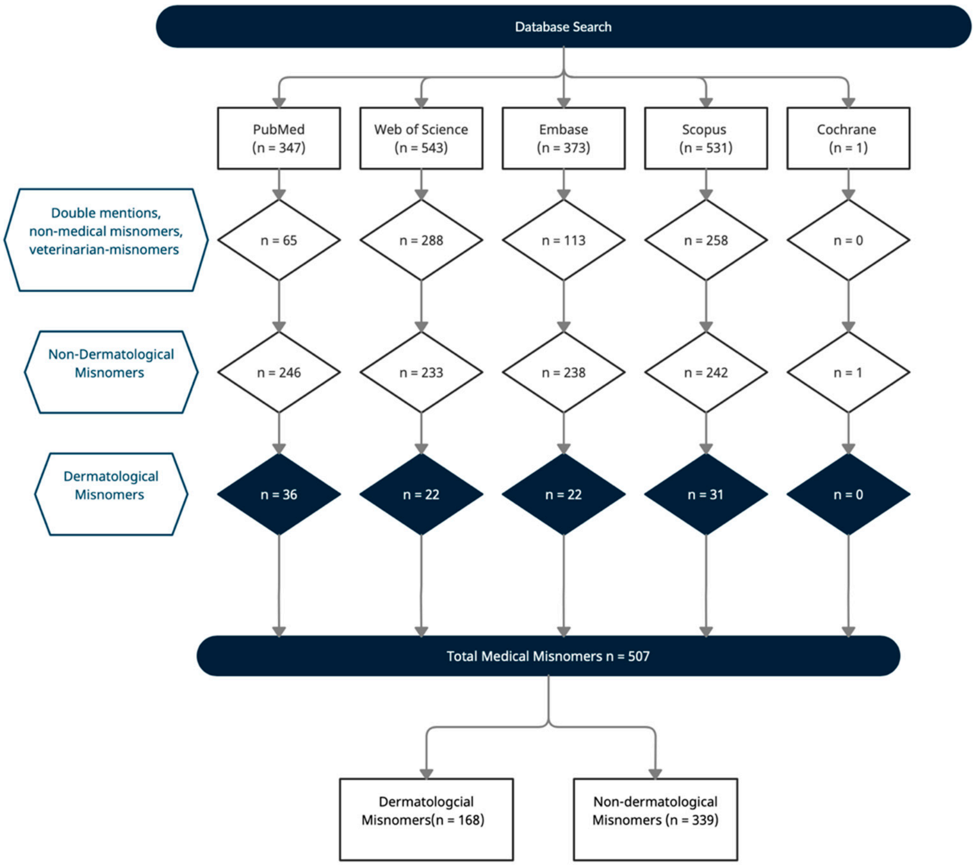

Figure S1: Flow diagram of the database search and study selection for the identification of medical misnomers.

Supplementary Text S3: German Misnomers (total n = 114)

1. (sub)- akute Proteinurie
2. Abklärung
3. Adulte Zystennieren
4. Alk negativ
5. Anpassungsstörung
6. Ataxie mit okulärer Apraxie
7. Bindungsstörung
8. Blinddarmentzündung
9. Blut-Hirn-Schranke
10. Borderline-Persönlichkeitsstörung
11. Breitbasige Diskusprotrusion
12. Bülau-Drainage
13. Cervicobrachialsyndrom
14. Wundverhältnisse
15. Diffuse Schmerzen
16. Digitalisieren
17. Dissoziale Persönlichkeitsstörung
18. Dose
19. Durchleuchtung
20. Elektrokrampftherapie
21. Endogene / Exogene Depression [4]
22. Entfaltungsknistern
23. Epiretinale Fibrose
24. Erkältung
25. Ertrinken
26. Fehlgeburt
27. Felsenbein
28. Fersensporn
29. Feuchte Gangrän
30. Feuchte Nebengeräusche
31. Fingerapoplex
32. Fixation
33. Flussbeschleunigung im LVOT
34. Freie intraabdominale Luft
35. Fruchtwasserembolie
36. Frühkindliche Psychose
37. Frühkindlicher Autismus
38. Goldstandard
39. Gürtelrose
40. Hallervorden-Spatz-Erkrankung
41. Hämorroidalthrombose
42. Herzenzyme
43. Hüftschnupfen
44. HWI
45. Hyperlaxizitätssyndrom
46. Idiopathischer Zehenspitzengang
47. Infarktpneumonie
48. Interruptio
49. Invasiver Harnwegsinfekt

50. Kernspintomographie
51. Knochendeckelreimplantation mit Palacos
52. Kollagenose
53. Konnektivitis
54. Konverison
55. Kopfgneis
56. Krampfleiden
57. Krebs
58. Laborchemisch
59. Linker Vorhof
60. Liquorleck
61. Luftleckage
62. Lumbago
63. Lungenembolie
64. Lymphdrüsenkrebs
65. Manisch depressiv
66. Marcoumarisieren
67. Milchunverträglichkeit
68. Minimal invasive Chirurgie
69. Mitralklappeninsuffizienz bei AVSD
70. Mohrenkopf
71. Mongolismus
72. Milchschorf
73. Multiorganversagen
74. Nackenfalte
75. Narkose
76. Narkosegas
77. Nicht-invasives papilläres Urothelkarzinom
78. Nierenversagen
79. Offset-Korrektur
80. Pilz-Vaginitis
81. Postaktinisch
82. Pseudokrupp
83. Pseudolymphom
84. Psychomotorischer Entwicklungsrückstand
85. Rechter Vorhof
86. Respirationsdefizit
87. Röntgen
88. Rückenmarkpunktion
89. Sakraldermoid
90. Sanfter Kaiserschnitt
91. Schenkelhalsfraktur
92. Schleudertrauma
93. Schreibaby
94. Schürfsaum (bei Schussverletzungen)
95. Schwangerschaftsvergiftung
96. Shaldon-Katheter
97. Silhouette-Zeichen
98. Spasmolytikum
99. Stimmband

100. Stressinkontinenz
101. Stützstrümpfe
102. Synkopieren
103. Teilnarkose
104. Terminieren
105. Trimalleolarfraktur
106. Trockene Nebengeräusche
107. Venflon
108. Verlangsamungsherd
109. Vesikuläratmen
110. Vollnarkose
111. Zentrale Facialisparese
112. Zwerchfellhernie
113. Zytostase
114. Kummerspeck [5]

Supplementary Text S4: French Misnomers (total n = 25)

1. Anti-maniaco-dépresseur
2. Anticalcique
3. Anticholestérol
4. Attaque
5. Au niveau de
6. Blocage
7. Cyphose pathologique
8. Décharge pseudomyotonique
9. Demi-vie
10. Effets secondaires
11. Facture de fatigue
12. Entérocolite nécrosante
13. Entérocolite post Hirschsprung
14. Fuite de protéines
15. Gran mal
16. Hernie étranglée
17. Luxation congénitale
18. Maladies des Membranes Hyalies
19. Pronation douloureuse
20. Proteinose alvéolaire
21. Résection antérieure basse
22. S´allumer
23. Taux plasmatique
24. Thrombose hémorroïdaire externe
25. Tumeur desmoïde

Supplementary Text S5: List of total dermatological misnomers (Total n = 166)

1. Wrong pathogenetic concepts: (Total n = 137, = 82.6%):

1. Acanthosis nigricans [2]
2. Acne [6]
3. Acne inversa [7]
4. Acne keloidalis [2]
5. Acne necrotica miliaris [8]
6. Actinic keratosis [2]
7. Adenoma sebaceum [2]
8. Colloid milium [2]
9. Alopecia mucinosa [8]
10. Anetoderma
11. Angioneurotic edema [1]
12. Arachnoidism [8]
13. Athlete's foot [2]
14. Atopic dermatitis [7]
15. Axillary freckling [8]
16. Botryomycosis [2]
17. Bullous congenital ichthyosiform erythroderma [9]
18. Candidiasis [1]
19. Capillaritis [9]
20. Cavernous hemangiomas [8]
21. Chloracne [10]
22. Chronic expanding hematoma [11]
23. Chronic superficial glossitis [8]
24. Collagen disorders or collagen vascular disorders [2]
25. Dermatitis factitia [6]
26. Dermatitis herpetiformis [6]
27. Dermatitis venata [6]
28. Dermoid cyst [12]
29. Dermatofibroma
30. Dissecting cellulitis of the scalp [8]
31. Dyshidrosis/dyshidrotic eczema [2]
32. Dysplastic melanocytic nevus [8]
33. Eccrine spiradenoma [8]
34. Eczema [6]
35. Eosinophilic cellulitis [13]
36. Epulis [1]
37. Eruptive syringoma [14]
38. Erysipela [6]
39. Erythema e pudore [13]
40. Erythema induratum [6]
41. Erythema migrans chronicum [8]
42. Erythema migrans arciforme et palpabile
43. Erythema multiforme [6]
44. Erythema toxicum neonatorum [2,8]
45. Fibroma [1]

46. Fibromatosis [1]
47. Follicular atrophoderma [2]
48. Gnaw warts [8]
49. Granuloma a(n)nulare [2]
50. Granuloma faciale [2]
51. Granuloma fissuratum [8,9]
52. Granuloma gluteale infantum [8]
53. Granuloma gravidarum [15]
54. Granuloma pyogenicum [13]
55. Granuloma teleangiectaticum [13]
56. Hemangioma [1]
57. Herpes gestationis [2]
58. Hidradenitis suppurativa [16]
59. Histiozytoma [13]
60. Histiozytosis X [17]
61. Horn cyst [2]
62. Hot-comb alopecia [2]
63. Invasive fungal infections [18]
64. Impetigo herpetiformis [2]
65. Insect bite [13]
66. Kaposi sarcoma [2]
67. Keratitis, ichthyosis and deafness (KID) syndrome [2,3]
68. Keratoderma blennorhagica [8]
69. Lentigo maligna [13]
70. Leukoedema [1]
71. Leukoplakia
72. Lichen [6]
73. Lichen planus [13]
74. Lichen ruber [13]
75. Lichen sclerosus [13]
76. Lichen scrophulosorum [6]
77. Lupus anticoagulant [8,19-21]
78. Lupus erythematosus [2]
79. Lupus pernio [22]
80. Lupus simplex [13]
81. Lymphangioma circumscriptum [8]
82. Lymphangiosarcoma [2]
83. Macrocephaly-Cutis Marmorata Telangiectatica Congenita Syndrome [22]
84. Malignant pustule of anthrax [2]
85. Melanocanthoma [8]
86. Moniliasis [2]
87. Mycosis fungoides [2]
88. Myxoid cyst [2]
89. Nasal glioma [8]
90. Necrobiotic Xanthogranuloma [8]
91. Necrolytic migratory erythema due to glucagonoma [23]
92. Neurodermitis [13]
93. Nevus cell nevus [13]
94. Nevus comedomicus [2]

95. Nevus depigmentosus [2]
96. (Nodulo)cystic acne [2]
97. Ocular albinism [8]
98. Palisaded encapsulated neuromas [2]
99. Parapsoriasis [13]
100. Pemphigus chronicus benignus familiaris [13]
101. Pemphigus neonatorum [2]
102. Peripheral ossifying fibroma [1]
103. Phakomatosis [2]
104. Piezogenic pedal papules [2]
105. Pilonidal sinus [2]
106. Pityriasis lichenoides acuta /chronica [13]
107. Pleomorphic adenoma / mixed tumor [1]
108. Porokeratosis [8]
109. Poroma [13]
110. Preauricular sinus [8]
111. Pretibial myxedema [2]
112. Proximal epithelioid sarcoma [24]
113. Pseudo-Kaposi sarcoma [13]
114. Psoriasis [2]
115. Pyoderma gangrenosum [2]
116. Ringworm [2,6]
117. Cutaneous sarcoidosis [2]
118. Scleredema [3]
119. Sebaceous cyst [25]
120. Seborrheic keratosis [2]
121. Seborrhoic dermatitis [2]
122. Siliconoma [2]
123. Sinusoidal hemangioma [2]
124. Squamous cell carcinoma [1]
125. Stasis dermatitis [3] / Stasis in venous ulcers [2,26]
126. Sycosis parasitica/vulgaris [6]
127. Tick bite [27]
128. Tinea amiantacea [2]
129. Tinea versicolor [2]
130. Transient acantholytic dermatosis [2]
131. Trichoadenoma (trichoadenoma of Nikolowski) [2]
132. Trichomycosis [2]
133. Uremic pruritus [28]
134. Urticaria pigmentosa [2]
135. Verrucous hemangioma [2]
136. Vulvar lichen sclerosus [29]
137. Zosteriform lichen planus [30]

2. Misleading word constructions: (Total n = 17, = 10.2%):

138. Actinomycetoma [8]
139. Basalioma [13]
140. Chloasma [2]
141. Congenital nevus [13]

142. Congenital triangular alopecia [2]
143. Cryolipolysis and Laser Lipolysis [31]
144. Focal dermal hypoplasia (Goltz syndrome) [2]
145. Juvenile xanthogranuloma [2]
146. Keratosis follicularis / Keratosis pilaris [2]
147. Lethal midline granuloma [32]
148. (Malignant) melanoma [1]
149. Mallorca acne [13]
150. Microdermabrasion [2]
151. Metastatic Crohn's [33]
152. Mycid [13]
153. Peruvian wart / verruga Peruana [8]
154. Rocky mountain spotted fever (RMSF) [2]

3. Mistranslations: (Total n = 2, = 1.2%):

155. Chicken pox [2]
156. Leprosy [2]

4. Analogies and Resemblances: (Total n = 5, = 3%):

157. Facies leontina
158. Hobnail hemangioma [2]
159. Kinky-hair disease [34]
160. Nevus flammeus [13]
161. Trumpeter's wart [8]

5. Eponyms: (Total n = 4, = 2.4%):

162. Fibroepithelioma of Pinkus [2]
163. Leser-Trélat sign [2,3]
164. Pautrier's microabscess [2]
165. Scleredema adultorum of Buschke [2]

6. Professional use of lay terms (Total n = 1, = 0.6%)

166. Knuckle pads [2]

7. Abbreviations (Total n = 0, = 0%)

Supplementary Text S6: List of dermatological misnomers from the database search (total n = 168)

1. Abscess Cavity [35]
2. Acanthosis nigricans [2]
3. Accessory scrotum or perineal collision-hamartoma [36]
4. Acne [6]
5. Acne inversa [37]
6. Acne keloidalis [2]
7. Acne necrotica miliaris [8]
8. Acne rosacea [2]
9. Acne urticaria [8]
10. Acne varioloformis [6]
11. Actinic keratosis [2]
12. Actinomycetoma [8]
13. Adenolymphoma [1]
14. Adenoma sebaceum [2]
15. Adult colloid milium [2]
16. Alopecia mucinosa [8]
17. Angioneurotic edema [1]
18. Arachnoidism [8]
19. Athlete's foot [2]
20. Atopic disease [7]
21. Auspitz phenomenon [3] / sign [2]
22. Axillary freckling [8]
23. Borst Jadassohn phenomenon [2]
24. Botryomycosis [2]
25. Bullous congenital ichthyosiform erythroderma [9]
26. Candidal milaria [8]
27. Candidiasis [1]
28. Capillaritis [9]
29. Cavernous hemangiomas [8]
30. Cerebellar haemangioblastoma [38]
31. Chemosurgery [2]
32. Chicken pox [2]
33. Chloasma [2]
34. Chloracne [10]
35. Chronic expanding hematoma [11]
36. Chronic superficial glossitis in syphilis [8]
37. Collagen disorders or collagen vascular disorders [2]
38. Congenital triangular alopecia [2]
39. Cortical desmoid [39]
40. Cosmetic dermatology versus cosmetology [40]
41. Cryolipolysis and Laser Lipolysis [31]
42. Dermatitis factitia [6]
43. Dermatitis herpetiformis [6]
44. Dermatitis medicamentosa [6]
45. Dermatitis papillaris capillitii [6]
46. Dermatitis repens [6]
47. Dermatitis venata [6]

48. Dissecting cellulitis of scalp [8]
49. Dyshidrosis and dyshidrotic eczema [2]
50. Dysplastic melanocytic nevus [8]
51. Eccrine spiradenoma [8]
52. Eczema [6]
53. Epulis [1]
54. Eruptive syringoma [14]
55. Erysipelas [6]
56. Erythema induratum [6]
57. Erythema migrans chronicum [8]
58. Erythema multiforme [6]
59. Erythema toxicum neonatorum [2,8]
60. Fibroepithelioma of Pinkus [2]
61. Fibroma [1]
62. Fibromatosis [1]
63. Focal dermal hypoplasia (Goltz syndrome) [2]
64. Follicular atrophoderma (Bazex-Dupré-Christol syndrome) [2]
65. Gnaw warts [8]
66. Granuloma annulare [2]
67. Granuloma faciale [2]
68. Granuloma fissuratum [8,9]
69. Granuloma gluteale infantum [8]
70. Granuloma gravidarum [15]
71. Hemangioma [1]
72. Herpes gestationis [2]
73. Hidradenitis suppurativa [16]
74. Histiozytosis X [17]
75. Hobnail hemangioma [2]
76. Horn cyst [2]
77. Hot-comb alopecia [2]
78. IFI = Invasive fungal infections [18]
79. Impetigo herpetiformis [2]
80. Infantile fibrosarcoma [41] / Infantile myofibromatosis [2]
81. Insect bite [13]
82. Juvenile xanthogranuloma [2]
83. Kaposi sarcom [2]
84. Keratitis, ichthyosis and deafness (KID) syndrome [2]
85. Keratoderma blennorhagica [8]
86. Keratosis follicularis [2] / Keratosis pilaris [2]
87. Kinky-hair disease [34]
88. Knuckle pads [2]
89. Leprosy [2]
90. Leser-Trelat sign [2,3]
91. Lethal midline granuloma [32]
92. Leukoedema [1]
93. Lichen [6]
94. Lichen scrophulosorum [6]
95. Lupus and lupoid [42]
96. Lupus anticoagulants [8,19-21]
97. Lupus erythematosus cell [2]

98. Lupus pernio [2]
99. Lymphangioma circumscriptum [8]
100. Lymphangiosarcoma [2]
101. Macrocephaly-Cutis Marmorata Telangiectatica Congenita Syndrome [22]
102. Malignant pustule of anthrax [2]
103. Melanoblastom [43]
104. Melanocanthoma [8]
105. Melanoma [1]
106. Metastatic Crohn's [33]
107. Microdermabrasion [2]
108. Mixed tumor of salivary tissue [44]
109. Moniliasis [2]
110. Mycosis fungoides [2]
111. Myxoid cyst [2]
112. Nasal glioma [8]
113. Necrobiotic Xanthogranuloma [8]
114. Necrolytic migratory erythema due to glucagonoma [23]
115. Nevus comedomicus [2]
116. Nevus depigmentosus [2]
117. Nodulocystic or cystic acne [2]
118. Ocular albinism [8]
119. Oral verrucous carcinoma [45]
120. Palisaded encapsulated neuromas [2]
121. Papular urticaria [2]
122. Pautrier's microabscess [2]
123. Pemphigus neonatorum [2]
124. Peripheral ossifying fibroma [1]
125. Peruvian wart / verruga Peruana [8]
126. Phakomatosis [2]
127. Piezogenic pedal papules [2]
128. Pilonidal sinus [2]
129. Pleomorphic adenoma / mixed tumor [1]
130. Porokeratosis [8]
131. Preauricular sinus [8]
132. Pretibial myxedema [2]
133. Proximal epithelioid sarcoma [24]
134. Psoriasis [2]
135. Pubic lice [2]
136. Pyoderma gangrenosum [2]
137. Ringworm [2,6]
138. Rocky mountain spotted fever (RMSF) [2]
139. Sarcoidosis [2]
140. Sarcoma [6]
141. Sclerdema [3]
142. Scleredema adultorum of Buschke [2]
143. Sebaceous cyst [25]
144. Seborrheic keratosis [2]
145. Seborrhoic dermatitis [2]
146. Siliconoma [2]

147. Sinusoidal hemangioma [2]
148. Soft wart [8]
149. Squamous cell carcinoma / epidermoid carcinoma [1]
150. Stasis dermatitis [3] / Stasis in venous ulcers [2,26]
151. Subungual malignant melanoma [46]
152. Sycosis parasitica [6]
153. Sycosis vulgaris [6]
154. Synovial sarcoma [8]
155. Ticks bite [27]
156. Tinea [2]
157. Tinea amiantacea [2]
158. Tinea cruris [8]
159. Tinea versicolor [2]
160. Transient acantholytic dermatosis [2]
161. Trichoadenoma (trichoadenoma of Nikolowski) [2]
162. Trichomycosis [2]
163. Trumpeter's wart [8]
164. Uremic pruritus [28]
165. Urticaria pigmentosa [2]
166. Verrucous hemangioma [2]
167. Vulvar lichen sclerosus [29]
168. Zosteriform lichen planus [30]

Supplementary Text S7: List of non-dermatological misnomers from the database search (total n = 339)

1. (Mid)face-lift [47,48]
2. (Skilled) Nursing homes [49,50]
3. 10-day rule [51]
4. 2-stage hypospadias repair [52]
5. 3-in-1 block [53]
6. Acalculous cholecystitis [54]
7. Accessory meningeal artery [55]
8. Acoustic neuroma [56]
9. Activating P14ARF-P53 for breast cancer therapy [57]
10. Acupuncture stimulation [58]
11. Acute epiglottitis [59]
12. Acute infectious nonbacterial gastroenteritis [60]
13. Adolescent sun protection at secondary school athletic sporting events [61]
14. Adrenal ovarian thecal metaplasia [62]
15. Agranulocytosis [1]
16. AIDS test [63]
17. Alkaline esophageal reflux [64]
18. Alveolar ejection volume [65]
19. Ameloblastoma [1]
20. Ampulla of Vater [66]
21. Andropause [67,68]
22. Anemia of chronic disease [69-73]
23. Angina with normal coronary arteries [74]
24. Ankyloglossia [1]
25. Anorexia nervosa [75]
26. Another Anesthetic disaster [76]
27. Anti-aging [77]
28. Antidepressants [78]
29. Aplastic anemia [79]
30. ASCUS [80]
31. Aseptic peritoneal cavity [81]
32. Aspiration pneumonia [82]
33. Asymptomatic hyperparathyroidism [83]
34. Asymptomatic intestinal malrotation [84]
35. Asymptomatic persistent or permanent atrial fibrillation [85]
36. Attention-deficit hyperactivity disorder [86]
37. Auriculotemporal neuralgia [1]
38. Autophagic cell death [87]
39. Bacterium melaninogenicum [88]
40. Basedow paraplegia [89]
41. Battered child [90]
42. Benign central neurocytoma [91]
43. Benign headache [92]
44. Benign monoclonal gammopathy [93]
45. Benign prostatic hyperplasia (BPH) [94]
46. Benign tertian malaria [95]

47. Benign tremulous Parkinson's disease [96]
48. Blood substitutes [97]
49. Blunt esophagectomy [98]
50. BM – the biggest misnomer in diabetes clinical practice [99]
51. Bone cyst [1]
52. Bordering on insanity [100]
53. Bronchial adenoma [101]
54. CA-125 [102]
55. CAL-PBM's [103]
56. Calcium agonist [104]
57. Calcium antagonist [104]
58. Campylobacter-pyloridis [105]
59. Cancer stem cells [106]
60. Canine hemifacial spasm [107]
61. Carcinoid [108,109]
62. Carcinoma in situ / intraepithelial carcinoma [1]
63. Cardiac asthma [110]
64. Casein kinase [111]
65. Caudal duplication [112]
66. Cavernous sinus [113]
67. Cerebral venous angioma [114]
68. Cesarean [115]
69. Choking game [116]
70. Chronic migraine [117]
71. Chronic trichinosis [118]
72. Clinical Hours in Nurse Practitioner Programs [119]
73. Clinical pharmacy [120]
74. Concussion [121]
75. Congenitally corrected transposition [122]
76. Contagion [123]
77. Conversion of the portal vein to an outflow tract [124]
78. Coronary Disease [125]
79. Coronary Lesion [125]
80. Cricoid pressure [126]
81. Crohn's disease [127]
82. CT stroke window settings [128]
83. Current bite mark analysis [129]
84. Cystourethroscopy [94]
85. Dead Tooth [130]
86. Deep tendon reflexes [131]
87. Deficit in dyslexia [132]
88. Demarcation membrane system of the megakaryocyte [133]
89. Dementia due to lacunar infarctions [134]
90. Dens in dente [1]
91. Dental phobia [135]
92. Diaphragmatic attenuation [136]
93. Diastolic heart failure [137]
94. Dilaceration [1]
95. Divergence insufficiency esotropia [138,139]
96. DMDx test for periodontits [140]

97. Dyskinesia of left ventricle [141]
98. Dysplasia [142]
99. Dysuria [94]
100. Early repolarization [143]
101. ECT [144]
102. Ectopia cloacae [145]
103. Ectopic ACTH secretion [146]
104. Elective ventilation [147]
105. Electric shock therapy [148]
106. Encapsulated papillary thyroid carcinoma, follicular variant [149]
107. Endometrioid carcinoma of the prostate [150]
108. Endoscopic papillotomy [151]
109. Endothelial dysfunction as a cause of erectile dysfunction [152]
110. ENT [153]
111. Eosinophilic granuloa [1]
112. Epigastric distress syndrome [154]
113. Epineurium on ultrasound [155]
114. Erector spinae transplant [156]
115. Euthyroid sick syndrome [157]
116. External hydrocephalus [158]
117. Extremity [131]
118. Fab-arm exchange [159]
119. Failed back syndrome [160]
120. False negative rate [161]
121. False positive exercise ECG [162]
122. Familial iris melanosis [163]
123. Familial Mediterranean fever [164]
124. Fanconi Anemia and Fanconi Syndrome [165]
125. Fetal pain [166]
126. Fibrositis [167]
127. Food safety and the hygiene [168]
128. Foramen of Monro [169]
129. Fourier transform lens [170]
130. Fractured penis [171]
131. Functional refractory period [172]
132. Garrè's osteomyelitis [1]
133. Gastroenteritis [173]
134. General secretory pathway [174]
135. Geriatric syndromes [175]
136. Germ-line gene therapy [176]
137. Germinal epithelium of the ovary [177]
138. Giggle micturition [178]
139. Gonorrheal spurs [179]
140. Granular cell myoblastoma [180]
141. Harderoporphyrin [181]
142. Healthy tan [182]
143. Hernia through the foramen of Bochdalek [183]
144. High blood pressure [184]
145. Highly active antiretroviral therapy [185]

146. Hot potato voice in peritonsillitis [186]
147. HTLV-1 and HIV-1 accessory proteins [187]
148. Human bovine arch [188,189]
149. Hyaline membrane syndrome or disease [190]
150. Hyalinizing trabecular adenoma [191]
151. Hygiene hypothesis for allergic disease [192]
152. Hyperchloraemic acidosis [193]
153. Hypoplastic left heart syndrome [194]
154. Iatrogenic [1]
155. Idarucizumab for Reversal of Dabigatran-Associated Bleeding [195]
156. Idiopathic infantile arterial calcification [196]
157. Idiopathic progressive chorea [197]
158. Immune reaction to smallpox vaccination [198]
159. Impulsivity [199]
160. Incomplete right bundle-branch block [200]
161. Instep flap [201]
162. Insulin neuritis [202]
163. Insulin resistance in diabetes mellitus in the African [203]
164. Intensive care unit syndrome [204]
165. International health [205]
166. International index of erectile function [206]
167. Interpolated premature ventricular contractions with postponed compensatory pauses [207]
168. Intracellular pH [208]
169. Intravascular bronchioloalveolar tumor [209]
170. Intravenous pylogram [94]
171. Iron Pill Gastritis [210]
172. Irritable bowel syndrom [211]
173. Isolated pulmonary valve stenosis [212]
174. Kummerspeck [5]
175. Laparoscopic sleeve gastrectomy [213]
176. Laser discectomy [214]
177. Latent carcinoma of the prostate [215]
178. Lateral aberrant thyroid [216]
179. Left atrial enlargement [217]
180. Localized mediastinal amyloidosis [218]
181. Long face [219]
182. Loose seton [220]
183. Lower Nephron nephrosis [221]
184. Lupus cerebritis [222]
185. Male climacteric [223]
186. Malignant external otitis [224]
187. Malignant hyperthermia [225]
188. Managed care [226]
189. Meconium aspiration syndrome [227]
190. Medically unexplained disability [228]
191. Mediterranean diet [229]
192. Menopausal [230]
193. Mentally retarded [231]

194. Mesopancreas [232]
195. Metabolically normal obesity [233]
196. Metaplastic sarcomatoid carcinoma of the breast with absent or minimal overt invasive carcinomatous component [234]
197. Metastatic ganglioneuroma [235]
198. Microscopic colitis [236]
199. Mild head injury [237]
200. Mild primary hyperparathyroidism [238]
201. Miller-Fisher Syndrome [239,240]
202. Morbi-RAS [241]
203. Morphine medicine [242]
204. Mucosa of the bladder and ureter [94]
205. Mustard gas keratitis [243]
206. Myositis ossificans [1]
207. Nervous dyspepsia [244]
208. Neurolemmoma [1]
209. Neuroleptic malignant syndrome [245]
210. New Delhi metallo beta lactamase-1 (NDM-1) [246]
211. Nil by mouth [247]
212. No Reflow Phenomenon [248]
213. Nodoventricular accessory connections [249]
214. Nonarteritic anterior ischemic optic neuropathy (NAION) [250-253]
215. Nonaversive behavior management [254]
216. Nondecolorized [255]
217. Obese (ob) gene [256]
218. Odontogenic [1]
219. Odontoma [1]
220. Oral-systemic link [257]
221. Os omovertebrale [258]
222. Osteitis [259]
223. Pale epiglottitis [260]
224. Palliative cytoreductive surgery and hyperthermic intraperitoneal chemoperfusion [261]
225. Pallidopyramidal diseaes [262]
226. Papworth cocktail [263]
227. Paradoxical embolism [264]
228. Paraplegia following epidural analgesia [265]
229. Paroxysmal atrioventricular block [266]
230. Percutaneous endoscopic duodenostomy [267]
231. Peripheral vasodilator drugs [268]
232. Pervasive refusal syndrome [269]
233. Physical medicine [270]
234. Pickwickian syndrome [271]
235. Pilonidal sinus or cyst [272]
236. Plastic and reconstructive surgery [273]
237. Pleurodesis for the treatment of primary spontaneous pneumothorax [274]
238. Plummer-Vinson syndrome [1]
239. Pneumocystis carinii vs. Pneumocystis jiroveci [275]

240. Polycystic ovarian syndrome [276,277]
241. Polymyalgia rheumatica [278]
242. Polysomy 17 by FISH for HER2 in breast cancer [279]
243. Post-stapedectomy reparative granuloma [280]
244. Post-tubal sterilization syndrome [281]
245. Posterior Reversible Encephalopathy [282]
246. Posterior tibial nerve [283]
247. Postobstructive pulmonary edema following hanging [284]
248. Postpartum psychosis [285]
249. Postperfusion lung syndrome [286]
250. Potency-sparing radical surgery [287]
251. Preemptive analgesia [288]
252. Prematurity [289]
253. Primary biliary cirrhosis [290,291]
254. Primary reading epilepsy [292]
255. Primary tropical splenic abscess [293]
256. Pseudomucinous cystadenoma [294]
257. Pterygomaxillary disjunction [295]
258. Pudendal block [296]
259. PUER (prostatectomy) [94]
260. Pulmonary veno-occlusive disease [297]
261. Pure obsessions [298]
262. Pyogenic granuloma of the larynx and trachea [299]
263. Radicular pain [131]
264. Rat bite fever [300]
265. Recurrent pilonidal disease [301]
266. Renal adenoma [302]
267. Renal resistive index [303]
268. Reperfusion injury [304]
269. Respirator lung [305]
270. Reverse cholesterol transport [306]
271. Reversible posterior leukoencephalopathy syndrome [307]
272. Richardson composite operation for uterine prolapse [308]
273. Riolan's arch [309]
274. Sclerosing hemangioma of the lung [209]
275. Secondary cartilage [310]
276. Secondary jaw aneurysmal bone cyst (JABC) [311]
277. Selective reduction [312]
278. Self-plagiarism [313,314]
279. Self-reinforcement [315]
280. Septic discitis [316]
281. Shaken baby syndrome [317]
282. Side effects [318]
283. Simple nephrectomy [319]
284. Single-Vessel disease / double-vessel disease / triple-vessel disease [125]
285. Skeletal-muscle spasm [320]
286. Slipped capital femoral epiphysis (SCFE) [321]
287. Slowly proliferating acute leukemia [322]
288. Species specificity of interferons [323]

289. Specific Reading Comprehension Disability [324]
290. Splenic artery steal syndrome [325]
291. Split syndrome [112]
292. Spontaneous sternal fracture [326]
293. Sporadic cretinism [327]
294. Stable intertrochanteric femoral fractures [328]
295. Stethoscope [329]
296. Stockholm syndrome manifestation of Munchausen [330]
297. Stroke [331]
298. Subclinical hypothyroidism [332]
299. Subcuticular suture [333]
300. Superficial femoral vein [334]
301. Superior oblique myokymia [335]
302. Symptoms of post-traumatic stress disorder in children with cancer and their parents [336]
303. Synovial sarcoma [337]
304. TAD [338]
305. Tendinitis [339]
306. Tenosynovitis in industry [340,341]
307. Teratomas in the currarino triad [342]
308. Thyromental distance and anterior larynx [343]
309. TMJ [344]
310. Tocopherol associated protein [345]
311. Total care [346]
312. Total mesorectal excision [347]
313. Traditional cell metaplasia of cervix [348]
314. Transient radicular irritation [349]
315. Transurethral [94]
316. Traumatic orchitis [350]
317. Traumatic unconsciousnes [351]
318. Treatment-resistant label in bipolar disorder [352]
319. Triple-negative breast cancer [353]
320. Trochanteric bursitis [354]
321. True agonadism [355]
322. Tubal ligation [356]
323. Tumor necrosis factor [357]
324. U wave [358]
325. Ultrastructure of an intermediate Sertoli-Leydig cell tumor [359]
326. Undifferentiated high-grade pleomorphic sarcoma (UHPS) [360]
327. Unverricht-Lundborg disease [361]
328. UVA-riboflavin collagen cross-linking [362]
329. Vaginal dermoid cyst [12]
330. Valsalva maneuver [363,364]
331. Valvular heart disease among non-valvular atrial fibrillation [365]
332. Venous angioma [366]
333. Venous thromboembolism as a never event [367]
334. Verrucous carcinoma of the larynx [368]

335. Viable but non-culturable and dormant bacteria [369]
336. Wave front correction [370]
337. White bile [371]
338. White-coat hypertension [372]
339. Y-Type Urethral Duplication [373]

Supplementary Text S8: List of dermatological misnomers from the questionnaire-based survey (total n = 65)

1. Acne inversa
2. Acne keloidalis nuchae
3. Actinomycetoma
4. Adenoma sebaceum
5. Angioedema
6. Basalioma
7. Botryomycosis
8. Chickenpox
9. Congenital nevus
10. Congenital temporal alopecia
11. Dermatitis herpetiformis
12. Dyshidrotic eczema
13. Dysplastic nevus
14. Eosinophilic cellulitis
15. Erythema e pudore
16. Granuloma faciale
17. Granuloma pyogenicum
18. Granuloma teleangiectaticum
19. Herpes gestationis
20. Hidradenitis suppurativa
21. Histiozytoma
22. Impetigo herpetiformis
23. Insect bite
24. Lentigo maligna
25. Lichen planus/ruber
26. Lichen sclerosus
27. Lupus antikoagulans
28. Lupus disseminatus faciei
29. Lupus miliaris faciei
30. Lupus pernio
31. Lupus vulgaris
32. Malignant Melanoma
33. Mallorca Acne
34. Molluscum pendulum
35. Mycosis fungoides
36. Mycid
37. Neurodermatitis

38. Nevus cell nevus
39. Nevus depigmentosus
40. Nevus flammeus
41. Parapsoriasis
42. Pautrier's microabscess
43. Pemphigus chronicus benignus familiaris
44. Pityriasis lichenoides acuta / chronica
45. Poroma
46. Pseudokaposi
47. Pseudopelade Brocq
48. Psoriasis pustulosa generalisata
49. Pyoderma gangraenosum
50. Ringworm
51. Scleroderma
52. Sebaceous cyst
53. Seborrhoeic keratosis
54. Sexual transmitted infection
55. Spider nevus
56. Spinalioma
57. Tick bite
58. Tinea amiantacea
59. Toxic epidermal nekrolysis
60. Trichomycosis axillaris
61. Trichomycosis palmelina
62. Ulcus molle
63. Urticaria pigmentosa
64. Xeroderma pigmentosum
65. Zystic hygroma

Supplementary Text S9: List of non-dermatological misnomers from the questionnaire-based survey (total n = 211)

1. Acoustic neuroma
2. Acute alcoholic hepatitis
3. Adipositas permagna
4. Agoraphobia
5. Alcoholism
6. Anaphylactic reaction
7. Anemia of chronic disease
8. Anesthesia
9. Aneurysma spurium
10. Aneurysmatic bone cyst
11. Angioma
12. Aniridia
13. Anorexia nervosa
14. Anterior Knee pain syndrom
15. Antibiosis
16. Antibiotic prophylaxis
17. Antiepileptics
18. Antihypertensiva
19. Anticoagulation
20. Antithrombin III
21. Aortic stenosis
22. Aplastic pancytopenia
23. Asphyxia
24. Aspiration pneumonia
25. Asthma bronchiale
26. Atheroma
27. Atypical chest pain
28. AV Channel
29. Bartholinitis
30. Benign papillary mesothelioma
31. Benign tremulous Parkinson's disease
32. Best supportive care
33. Blephalorchalasis
34. Bovine Arch
35. Bronchopulmonary dysplasia
36. Bursitis trochanterica
37. Carcinoma in situ
38. Carzinoid
39. CCAM
40. Chemical castration
41. Chemotherapy
42. Chondrocalcinosis
43. Chondropathia patellae
44. Chronic Bronchitis
45. Chronic lyme disease
46. Chronic lymphocytic leukemia
47. Chronic Sinusitis

48. Churg-Strauss-Syndrom
49. Clara cells
50. Collapse
51. Colon
52. Comfort therapy
53. Compliance
54. Cormack
55. CUP Syndrom
56. CVI
57. Cystic fibrosis
58. Delirium tremens
59. Diastolic heart failure
60. Diastolic steal
61. DIC
62. Diplegia
63. Down Syndrom
64. Ductus botalli
65. Ectopia cordis
66. Endoleak Typ V
67. Epicondylitis
68. ER positiv
69. ERCP
70. Erythropoesis
71. Fagerström Test for Nicotine Dependence
72. Fail Chest
73. FDG-active
74. Febrile Neutropenia
75. Fibromyalgia
76. Fibrous dysplasia
77. Focal Nephritis
78. FUO
79. Gastroschisis
80. Gentamycin
81. Gestational diabetes
82. Gestose
83. Glaukoma chronicum simplex
84. Glioblastoma
85. Glucose intolerance
86. Gonorrhoe
87. Hematoma
88. Hemifacial spasm
89. Hemiplegia
90. Hepatojugular reflux
91. Horner Syndrom
92. Hyaline membrance snydrome or disease
93. Hyaline membranes
94. Hydrops cochleae
95. Hypostatic pneumonia
96. Immunotherapy
97. Impingement Syndrom

98. Impuls control disorder in M. Parkinson
99. Incarcerated hernia
100. Infection urinaire
101. Infect parameter
102. Inflammatory aneurysm
103. Interstitial lung diesease
104. Iron deficiency
105. Irritable bowel syndrom
106. Jones fracture
107. Jump graft
108. Kanner Syndrome
109. Kippel-Trenauany-Syndrome
110. Legionnaire's disease
111. Leukemia
112. Limited cutaneoius systemic sclerosis
113. Lymphangioma
114. Malaria
115. Malignant Hyperthermia
116. Mastopathy
117. Meningitis
118. Mesothelioma in situ
119. Metabolic syndrome
120. Midline Granuloma
121. Morbus Bowen
122. Morbus Sudeck
123. Morbus Wegener
124. Morbus Weil
125. Morton' Neurinoma
126. Mukoviszidosis
127. Multiple Myeloma
128. Multiple Sklerosis
129. Mycotic aneurysm
130. Myelodysplastic syndrome
131. Myeloma kidney
132. Myogelosis
133. Nasal glioma
134. Necrotizing pneumonia
135. Neurasthenia
136. Neuroleptique
137. Neuronitis vestibularis
138. Neurotic disorder
139. Non-fatty liver disease
140. Occult bacteremia
141. Optic Glioma
142. Organising pneumonia
143. Osteoarthrosis
144. Osteochondrosis dissecans
145. Osteoma
146. Palliativ
147. Pancoast Tumor

148. Paradoxical diahhrea
149. Parasympatholytic
150. Perfusor
151. Peritonitis
152. Pernicious anemia
153. Personality disorder
154. Pes equinovarus
155. Phlebitis
156. Phyllodes Tumor
157. Pigmented villododulr synovitis
158. Polytoxycomania
159. Primary billiary cirrhosis
160. Pseudotumor cerebri
161. Pseudotumor orbitae
162. Pulmonal-arterial hypertonia (PAH)
163. Pulmonary edema
164. Pulmonary Hamartoma
165. Pulsus paradoxus
166. Q fever
167. Radiology
168. Radiosurgery
169. Recruitment
170. Reflux nephropathy
171. Retinitis pigmentosa
172. Retinitis serosa
173. Rhinopathy
174. Sarkoidosis
175. Schizophrenia
176. Secondary hyperparathyroeoidism
177. Sepsis
178. Septic arthritis
179. Seroma
180. SI-SII-SIII-Type
181. Side effect
182. Sigmoidectomy
183. Sinus vein thrombosis
184. Sinusitis
185. Spondylarthropathy
186. Stabilisation
187. Stroke
188. Subclinical hypothryoidism
189. Superficial phlebitis
190. Synovial sarcoma
191. Systolic heart failure
192. Tennis Elbow
193. Testicular cancer
194. Testicular torsion
195. Thrombopenia
196. Thrombophlebitis
197. Tourette Syndrom

198. Toxidermy
199. Trauma X
200. TVT
201. Uremic pruritus
202. Vasovagale Syncope
203. Vena femoralis superficialis
204. Vestibulitis
205. Vital parameters
206. Vitamin D
207. Vitamin D deficiency
208. Wedge resection
209. Wegener-Vaskulitis
210. Wet lung
211. Whipples operation

Supplementary Text S10: Introduction of unreported misnomers collected in the questionnaire-based survey

Table S1: Unreported misnomers collected in the questionnaire-based survey, including explanations and suggested terminology.

Misnomer	Explanation	Suggestion for improvement
**1. Basalioma**	Ending suggests a benign disease	Basal cell carcinoma
**2. Congenital nevus**	Is neither hereditary nor present at birth	Infantile nevus
**3. Eosinophilic cellulitis**	Not an infection of the subcutis but the dermis	Eosinophilic dermatitis
**4. Erythema e pudore**	Not only triggered by shame, but by any involuntary flushing associated with emotions	Emotional erythema
**5. Granuloma telangiectaticum**	Not granulomatous	Reactive vascular proliferation syn-drome, telangiectatic type
**6. Histiocytoma**	Not part of histiocytes but cells of the dermis	Dermatofibroma
**7. Insect bite**	Insects don’t sting, they bite	Insect sting
**8. Lentigo maligna**	Lentigo suggests a benign disesae	Melanoma in situ
**9. Lichen ruber/planus**	Purely descriptive, does not capture pathophysiology;	No suggestion made

	moreover "ruber" and "planus" may not be appropriate as other colors and elevated/hyperkeratotic forms exist	
**10. Lichen sclerosus**	Purely descriptive, does not capture pathophysiology	No suggestion made
**11. Malignant melanoma**	The suffix "-oma" suggests benignity. The addition of the adjective "malign" to melanoma (common in Germanspeaking areas) is also misleading because it suggests the existence of a "benign" melanoma	Melanoma or more precisely melanocytic malignancy
**12. Mallorca acne**	Does not occur only in Mallorca	Suncream/bath oil acne
**13. Mycid**	Does not reflect the clinical presentation and	Hyperergic reaction to dermatophytes
**14. Neurodermatitis/Neurodermitis**	Not a primary disease of skin nerves	Atopic dermatitis
**15. Parapsoriasis**	Purely descriptive term which may be misleading	Cutaneous T-cell lymphoma early stage
**16. Pemphigus chronicus benignus familiaris Hailey-Hailey**	Eponym, moreover it is not pemphigus/an	Acantholytic dermatitis due to

	autoimmune bullous disease	ATP2C1 gene mutation
**17. Pityriasis lichenoides acuta /chronica**	Purely descriptive, does not reflect the underlying pathophysiology	No suggestion made
**18. Porocarcinoma**	There are no "poro cells"	Carcinoma of poroid cells
**19. Pseudo-Kaposi sarcoma**	Is not a sarcoma	Reactive angiodysplasia of cutaneous blood vessels

References

1. Subramanyam, R.V. Misnomers in oral pathology. *Oral Dis* **2010**, *16*, 740–746, doi:10.1111/j.1601-0825.2010.01695.x.
2. Hulmani, M.; Kudur, M. Misnomers in dermatology: time to change and update. *Indian J Dermatol Venereol Leprol* **2013**, *79*, 479–491, doi:10.4103/0378-6323.113075.
3. Barankin, B.; Freiman, A. Misnomers in dermatology. *J Cutan Med Surg* **2005**, *9*, 284–288, doi:10.1007/s10227-005-0102-7.
4. Skeat, W. *Concise Dictionary of English Etymology: the pioneering work on the roots and origins of English language*; Wordsworth Editions Ltd: London, UK, 1993.
5. van Strien, T.; Donker, M.H.; Ouwens, M.A. Is desire to eat in response to positive emotions an 'obese' eating style: Is Kummerspeck for some people a misnomer? *Appetite* **2016**, *100*, 225–235, doi:10.1016/j.appet.2016.02.035.
6. Scholtz, M. Dermatologic Misnomers. *Arch Term Syphilol* **1920**, *1*, 182–190.
7. Akdis, C.A.; Ballas, Z.K. Atopic disease is a misnomer. *J. Allergy Clin. Immunol.* **2016**, *137*, 699, doi:10.1016/j.jaci.2016.01.033.
8. Savitha, S.A.; Sacchidanand, S.A.; Gowda, S.K. Misnomers in dermatology: an update. *Indian J Dermatol* **2013**, *58*, 467–474, doi:10.4103/0019-5154.119962.
9. Nosrati, N.; Harting, M.S.; Yang, D.J.; Shen, Y.A.; Maender, J.L.; Jogi, R.P.; Sonabend, M.L.; Hsu, S. Dermatology misnomers. *Dermatol Online J* **2008**, *14*, 22.
10. Saurat, J.H.; Sorg, O. Chloracne, a misnomer and its implications. *Dermatology* **2010**, *221*, 23–26, doi:10.1159/000290672.
11. Ehara, S. Chronic expanding hematoma: mystery or misnomer? *AJR Am J Roentgenol* **2004**, *183*, 243–244, doi:10.2214/ajr.183.1.1830243.
12. Siu, S.S.; Tam, W.H.; To, K.F.; Yuen, P.M. Is vaginal dermoid cyst a rare occurrence or a misnomer? A case report and review of the literature. *Ultrasound Obstet Gynecol* **2003**, *21*, 404–406, doi:10.1002/uog.97.
13. Griss, V. Misnomers in Medicine: Assessment of the approximative number and relevance of misnomers in dermatology and other medical specialties. Master's Thesis, University of Basel, Basel, Switzerland, 2016.
14. Guitart, J.; Rosenbaum, M.M.; Requena, L. 'Eruptive syringoma': a misnomer for a reactive eccrine gland ductal proliferation? *J Cutan Pathol* **2003**, *30*, 202–205, doi:10.1034/j.1600-0560.2003.00023.x.
15. Danesh, M.; Pomeranz, M.K.; McMeniman, E.; Murase, J.E. Dermatoses of pregnancy: Nomenclature, misnomers, and myths. *Clin Dermatol* **2016**, *34*, 314–319, doi:10.1016/j.clindermatol.2016.02.002.

16. Sellheyer, K.; Krahl, D. "Hidradenitis suppurativa" is acne inversa! An appeal to (finally) abandon a misnomer. *Int J Dermatol* **2005**, *44*, 535–540, doi:10.1111/j.1365-4632.2004.02536.x.
17. Teplitz, C.; Goss, G. Histiocytosis X—Misnomer in systemic Langerhans cell proliferative disorders: ultrastructure of distinctive infiltrative cell and morphogenesis of its pathognomonic granules. 1975; pp. 2110–2118.
18. Hof, H. IFI = invasive fungal infections. What is that? A misnomer, because a non-invasive fungal infection does not exist! *Int J Infect Dis* **2010**, *14*, e458–459, doi:10.1016/j.ijid.2009.08.006.
19. Wong, R.W.; Chan, J.K.; Wong, K.L. Lupus anticoagulant--a double misnomer. *Asian Pac J Allergy Immunol* **1987**, *5*, 161–165.
20. Golbus, J.; Fox, D.A. The lupus anticoagulant: a confusing misnomer. *Ann Intern Med* **1987**, *106*, 911, doi:10.7326/0003-4819-106-6-911_1.
21. Triplett, D.A.; Brandt, J.T. Lupus anticoagulants: misnomer, paradox, riddle, epiphenomenon. *Hematol Pathol* **1988**, *2*, 121–143.
22. Wright, D.R.; Frieden, I.J.; Orlow, S.J.; Shin, H.T.; Chamlin, S.; Schaffer, J.V.; Paller, A.S. The misnomer "macrocephaly-cutis marmorata telangiectatica congenita syndrome": report of 12 new cases and support for revising the name to macrocephaly-capillary malformations. *Arch Dermatol* **2009**, *145*, 287–293, doi:10.1001/archdermatol.2008.545.
23. Grewal, P.; Salopek, T.G. Is necrolytic migratory erythema due to glucagonoma a misnomer? A more apt name might be mucosal and intertriginous erosive dermatitis. *J Cutan Med Surg* **2012**, *16*, 76–82, doi:10.2310/7750.2011.10134.
24. Sur, M.; Nayler, S.J. Proximal epithelioid sarcoma--a misnomer. *Histopathology* **2001**, *39*, 641–643, doi:10.1046/j.1365-2559.2001.1311d.x.
25. Parkash, S.; Chandrasekaran, V. Sebaceous cyst--a misnomer. *J Indian Med Assoc* **1982**, *79*, 29–31.
26. Schwartzberg, J.B.; Kirsner, R.S. Stasis in venous ulcers: a misnomer that should be abandoned. *Dermatol Surg* **2000**, *26*, 683–684, doi:10.1046/j.1524-4725.2000.00026.x.
27. Löser, C.; Mehlhorn, H.; Schill, W.B. The tick stings! Observations on a misnomer. *Hautarzt* **2002**, *53*, 91–92.
28. Saxena, A.K. "Uremic pruritus": a misnomer. *Hemodial Int* **2005**, *9*, 416–417, doi:10.1111/j.1542-4758.2005.01161.x.
29. Parra, E. Vulvar lichen sclerosus: A misnomer for an entity with decreased fibrillar components and increased amorphous components in extracellular matrix remodeling. 2012; p. S324.
30. Happle, R. "Zosteriform" lichen planus: the bizarre consequences of a misnomer. *Acta Derm Venereol* **1998**, *78*, 300, doi:10.1080/000155598441936.
31. Friedmann, D.P.; Mishra, V. Cryolipolysis and Laser Lipolysis: Misnomers in Cosmetic Dermatology. *Dermatol Surg* **2015**, *41*, 1327–1328, doi:10.1097/dss.0000000000000474.
32. Lotan, A.; Eliachar, I.; Joachims, H.Z.; Lichtig, H. Lethal midline granuloma: a misnomer. *Harefuah* **1977**, *92*, 563–565.
33. Anadolu, R.; Calikoğlu, E.; Karayalçin, S.; Gürgey, E. Cutaneous Crohn's disease: 'metastatic Crohn's is a misnomer'. *J Eur Acad Dermatol Venereol* **1999**, *13*, 67–68, doi:10.1111/j.1468-3083.1999.tb00850.x.
34. Powell, J.; Ferguson, D.J.; Dawber, R.P. Is kinky-hair disease a misnomer for Menkes syndrome? *Arch Dermatol* **2001**, *137*, 92–93, doi:10.1001/archderm.137.1.92.
35. Dias, G.; Tayles, N. 'Abscess cavity'—a misnomer. *Int. J. Osteoarchaeol.* **1997**, *7*, 548–554, doi:10.1002/(SICI)1099-1212(199709/10)7:5<548::AID-OA362>3.0.CO;2-5.
36. Amann, G.; Berger, A.; Rokitansky, A. Accessory scrotum or perineal collision-hamartoma. A case report to illustrate a misnomer. *Pathol Res Pract* **1996**, *192*, 1039–1043; , doi:10.1016/s0344-0338(96)80046-x.
37. Boer, J.; Nazary, M. Long-term results of acitretin therapy for hidradenitis suppurativa. Is acne inversa also a misnomer? *Br J Dermatol* **2011**, *164*, 170–175, doi:10.1111/j.1365-2133.2010.10071.x.
38. Cerebellar haemangioblastoma a misnomer. *Lancet* **1967**, *1*, 1095–1096, doi:10.1016/S0140-6736(67)92155-4.
39. Hall, F.M. Cortical desmoid: a misnomer? *AJR Am J Roentgenol* **2011**, *197*, 1022, doi:10.2214/ajr.11.6842.
40. Verma, S.B.; Draelos, Z.D. Cosmetic dermatology versus cosmetology: a misnomer in need of urgent correction. *Indian J Dermatol Venereol Leprol* **2008**, *74*, 92–93, doi:10.4103/0378-6323.39687.
41. Wilson, M.B.; Stanley, W.; Sens, D.; Garvin, A.J. Infantile fibrosarcoma--a misnomer? *Pediatr Pathol* **1990**, *10*, 901–907, doi:10.3109/15513819009064725.
42. Pourazizi, M.; Kabiri, S.; Saffaei, A.; Abtahi-Naeini, B. Lupus and lupoid misnomer: issues and challenges. *Lupus* **2017**, *26*, 6–9, doi:10.1177/0961203316665711.
43. Cramer, S.F. Melanoblastoma is a misnomer. *Pediatr Pathol Lab Med* **1997**, *17*, 532–534, doi:10.1080/15513819709168595.

44. Lund, C.C.; Lee, F.B.; Campagna-Pinto, D.F. Mixed tumor of salivary tissue; a harmful misnomer for a carcinoma. *Am Surg* **1958**, *24*, 747–752.
45. Ray, J.G.; Mukherjee, S.; Pattanayak Mohanty, S.; Chaudhuri, K. Oral verrucous carcinoma--a misnomer? Immunohistochemistry based comparative study of two cases. *BMJ Case Rep* **2011**, *2011*, doi:10.1136/bcr.11.2010.3479.
46. Saida, T. Heterogeneity of the site of origin of malignant melanoma in ungual areas: 'subungual' malignant melanoma may be a misnomer. *Br J Dermatol* **1992**, *126*, 529, doi:10.1111/j.1365-2133.1992.tb15134.x.
47. McCollough, E.G.; Scurry, W.C., Jr.; Shirazi, M.A. The "midface-lift" as a misnomer for correctly identifying procedures designed to lift and rejuvenate the cheeks and malar regions of the face. *Arch Facial Plast Surg* **2009**, *11*, 257–262, doi:10.1001/archfacial.2009.50.
48. Maguire, C.P. The face lift - a misnomer. *J S C Med Assoc* **1980**, *76*, 377–378.
49. Conant, S.M. Is 'skilled nursing home' a misnomer? Least educated providers constitute largest proportion of workforce. *Am J Nurs* **2004**, *104*, 11, doi:10.1097/00000446-200406000-00002.
50. Kennedy, M.S. 'Nursing homes': a misnomer. *Am J Nurs* **2014**, *114*, 7, doi:10.1097/01.NAJ.0000456406.24376.9a.
51. Mole, R.H. The 10-day rule: a misnomer. *Radiography* **1984**, *50*, 229–230.
52. Shukla, A.R.; Patel, R.P.; Canning, D.A. The 2-stage hypospadias repair. Is it a misnomer? *J Urol* **2004**, *172*, 1714–1716, doi:10.1097/01.ju.0000138926.26530.f9.
53. Flores, R.A., Jr. 3-in-1 block: are we still using this misnomer? *AANA J* **2013**, *81*, 171.
54. Barrett, D.S.; Chadwick, S.J.; Fleming, J.A. Acalculous cholecystitis--a misnomer. *J R Soc Med* **1988**, *81*, 664, doi:10.1177/014107688808101117.
55. Vitek, J.J. Accessory meningeal artery: an anatomic misnomer. *AJNR Am J Neuroradiol* **1989**, *10*, 569–573.
56. Pineda, A.; Feder, B.H. Acoustic neuroma: a misnomer. *Am Surg* **1967**, *33*, 40–43.
57. Hatoum, D.; Martiniello-Wilks, R.; Nassif, N.; Yagoub, D.; McGowan, E.M. THE MISNOMER OF ACTIVATING P14ARF-P53 FOR BREAST CANCER THERAPY. *J. Gene Med.* **2013**, *15*, 333–334.
58. Silberstein, M. Is acupuncture "stimulation" a misnomer? A case for using the term "blockade". *BMC Complement Altern Med* **2013**, *13*, 68, doi:10.1186/1472-6882-13-68.
59. Woo, J.K.; van Hasselt, C.A. Acute epiglottitis: a misnomer. *Otolaryngol Head Neck Surg* **1994**, *111*, 538–539, doi:10.1177/019459989411100427.
60. Widerlite, L.; Trier, J.S.; Blacklow, N.R.; Schreiber, D.S. ACUTE INFECTIOUS NONBACTERIAL GASTROENTERITIS - MISNOMER. *Gastroenterology* **1974**, *66*, 799.
61. McNoe, B.M.; Reeder, A.I. Adolescent sun protection at secondary school athletic sporting events - a misnomer. *Aust N Z J Public Health* **2016**, *40*, 313–315, doi:10.1111/1753-6405.12512.
62. Mete, O.; Raphael, S.; Pirzada, A.; Asa, S.L. Is adrenal ovarian thecal metaplasia a misnomer? Report of three cases of radial scar-like spindle cell myofibroblastic nodule of the adrenal gland. *Endocr Pathol* **2011**, *22*, 222–225, doi:10.1007/s12022-011-9175-6.
63. Pinching, A.J. "AIDS" test a misnomer. *Br Med J (Clin Res Ed)* **1985**, *291*, 821, doi:10.1136/bmj.291.6498.821.
64. Singh, S.; Richter, J.E. Alkaline esophageal reflux--a misnomer. *Gastroenterology* **1993**, *104*, 947–949, doi:10.1016/0016-5085(93)91049-n.
65. Fletcher, R.; Drummond, G.B. Alveolar ejection volume: a misnomer? *Eur Respir J* **2000**, *15*, 232–233, doi:10.1034/j.1399-3003.2000.15a41.x.
66. Suárez, C.V. Ampulla of Vater-a misnomer. *Mt Sinai J Med* **1980**, *47*, 373–385.
67. Tan, R. Re: andropause: a misnomer for a true clinical entity. *J Urol* **2000**, *164*, 1319.
68. Morales, A.; Heaton, J.P.; Carson, C.C., 3rd. Andropause: a misnomer for a true clinical entity. *J Urol* **2000**, *163*, 705–712, doi:10.1016/s0022-5347(05)67788-9.
69. Kushner, I. Anemia of chronic disease: a misnomer? *Ann Intern Med* **1992**, *116*, 521.
70. Vreugdenhil, G.; Swaak, A.J. Anemia of chronic disease: a misnomer? *Ann Intern Med* **1992**, *116*, 520–521.
71. Haurani, F.I. Anemia of chronic disease: a misnomer? *Ann Intern Med* **1992**, *116*, 520.
72. Fuchs, D.; Weiss, D.; Wachter, H. Anemia of chronic disease: a misnomer? *Ann Intern Med* **1992**, *116*, 520.
73. Schilling, R.F. Anemia of chronic disease: a misnomer. *Ann Intern Med* **1991**, *115*, 572–573, doi:10.7326/0003-4819-115-7-572.
74. Ruwitch, J.R., Jr.; Ludbrook, P.A.; Sobel, B.E. Angina with "normal coronary arteries". A misnomer. *Adv Cardiol* **1977**, *20*, 27–40, doi:10.1159/000399851.
75. Lask, B.; Frampton, I. Anorexia nervosa--irony, misnomer and paradox. *Eur Eat Disord Rev* **2009**, *17*, 165–168, doi:10.1002/erv.933.
76. Bickford-Smith, P.; Cook, P.; Green, M. Another anesthetic disaster: a misnomer. *Anaesthesia* **1985**, *40*, 502.
77. Bernard, R.W. "Anti-aging": a misnomer? *Aesthet Surg J* **2002**, *22*, 456–457, doi:10.1067/maj.2002.128627.

78. Sparhawk, R. In bipolar disorder beyond 10 weeks of treatment, the term antidepressants is a misnomer. *J Clin Psychiatry* **2011**, *72*, 871, doi:10.4088/JCP.11l06953.
79. Stavem, P. Aplastic pancytopenia instead of the misnomer aplastic anaemia. *Br. J. Haematol.* **1991**, *77*, 258a.
80. Cashmore, R.W. ASCUS: a misnomer. *Acta Cytol* **1997**, *41*, 1864–1865.
81. Roberts, K.; Johnson, W.W.; Bruckner, H.S. The aseptic peritoneal cavity- A misnomer. *Surg. Gynecol. Obstet.* **1933**, *57*, 752–761.
82. Jurado, R.L.; Franco-Paredes, C. Aspiration pneumonia: a misnomer. *Clin Infect Dis* **2001**, *33*, 1612–1613, doi:10.1086/323016.
83. Perrier, N.D. Asymptomatic hyperparathyroidism: a medical misnomer? *Surgery* **2005**, *137*, 127–131, doi:10.1016/j.surg.2004.06.037.
84. Cohen, Z.; Kleiner, O.; Finaly, R.; Mordehai, J.; Newman, N.; Kurtzbart, E.; Mares, A.J. How much of a misnomer is "asymptomatic" intestinal malrotation? *Isr Med Assoc J* **2003**, *5*, 172–174.
85. Ganapathy, A.V.; Monjazeb, S.; Ganapathy, K.S.; Shanoon, F.; Razavi, M. "Asymptomatic" persistent or permanent atrial fibrillation: A misnomer in selected patients. *Int J Cardiol* **2015**, *185*, 112–113, doi:10.1016/j.ijcard.2015.03.122.
86. Wasserman, T.; Wasserman, L.D. The misnomer of attention-deficit hyperactivity disorder. *Appl Neuropsychol Child* **2015**, *4*, 116–122, doi:10.1080/21622965.2015.1005487.
87. Kroemer, G.; Levine, B. Autophagic cell death: the story of a misnomer. *Nat Rev Mol Cell Biol* **2008**, *9*, 1004–1010, doi:10.1038/nrm2529.
88. Schwabacher, H.; Lucas, D.R.; Rimington, C. Bacterium melaninogenicum; a misnomer. *J Gen Microbiol* **1947**, *1*, 109–120, doi:10.1099/00221287-1-2-109.
89. Smith, L.; Kemp, T.; van der Meyden, C.H.; Schutte, C.M. Basedow paraplegia: A possible misnomer. *S Afr Med J* **2015**, *105*, 875, doi:10.7196/samjnew.8768.
90. Lenoski, E.F. The battered child a misnomer? *J. Am. Coll. Emerg. Physicians* **1973**, *2*, 101–102.
91. Schild, S.E. Benign central neurocytoma: a double misnomer? *Cancer* **2002**, *94*, 284, doi:10.1002/cncr.10183.
92. Jones, J.M. Benign headache . . . A possible misnomer? *Headache* **2009**, *49*, 1120, doi:10.1111/j.1526-4610.2009.01467.x.
93. Kyle, R.A. 'Benign' monoclonal gammopathy. A misnomer? *JAMA* **1984**, *251*, 1849–1854.
94. Douglas, L.L. Urologic terminology and misnomers. *Urology* **1983**, *22*, 98, doi:10.1016/0090-4295(83)90364-3.
95. Bammigatti, C.; Shetty, S.; Shetty, S.; Kumar, A. Benign tertian malaria--a misnomer? *Trop Doct* **2011**, *41*, 168–169, doi:10.1258/td.2011.110025.
96. Deuschl, G. Benign tremulous Parkinson's disease: a misnomer? *Mov Disord* **2013**, *28*, 117–119, doi:10.1002/mds.25317.
97. Blajchman, M.A. Blood substitutes--a misnomer, nevertheless a promise. *Can J Surg* **1982**, *25*, 361–362.
98. Kron, I.L.; Joob, A.; Johns, M.E.; Minor, G.R. Blunt esophagectomy: a misnomer. *Va Med* **1984**, *111*, 216–217.
99. Bannister, M. "BM" - The biggest misnomer in diabetes clinical practice. *J. Diabetes Nurs.* **2013**, *17*, 46.
100. Warne, T.; McAndrew, S. Bordering on insanity: misnomer, reviewing the case of condemned women. *J Psychiatr Ment Health Nurs* **2007**, *14*, 155–162, doi:10.1111/j.1365-2850.2007.01058.x.
101. Rowe, L.D.; Jafek, B.W. Bronchial adenoma: a malignant misnomer. *Laryngoscope* **1979**, *89*, 1991–1999, doi:10.1288/00005537-197912000-00012.
102. Bansal, A.; Gupta, R.; Prakash, A.; Jyala, N.S. CA-125, just a tumor marker: A misnomer. *Indian J. Gastroenterol.* **2015**, *34*, A60.
103. Grant, J.M. The CAL-PBM's a misnomer? *Aust. J. Audiol.* **1980**, *2*, 19–21.
104. Weishaar, R.E. "Calcium antagonists" and "calcium agonists": is there a place in pharmacology for these two misnomers? *Life Sci* **1984**, *35*, 455–462, doi:10.1016/0024-3205(84)90237-6.
105. Leonbarua, R.; Spira, W.M.; Ramirezramos, A.; Gilman, R.H.; Recavarren, S.; Watanabe, J. CAMPYLOBACTER-PYLORIDIS - A COMPLETE MISNOMER. *Gastroenterology* **1987**, *92*, 1501.
106. Maenhaut, C.; Dumont, J.E.; Roger, P.P.; van Staveren, W.C. Cancer stem cells: a reality, a myth, a fuzzy concept or a misnomer? An analysis. *Carcinogenesis* **2010**, *31*, 149–158, doi:10.1093/carcin/bgp259.
107. Motta, L.; de Lahunta, A. Canine hemifacial spasm: a misnomer? *J Small Anim Pract* **2015**, *56*, 480, doi:10.1111/jsap.12376.
108. Soga, J. [Reevaluation of the term "carcinoid"--how to deal with this misnomer]. *Nihon Rinsho* **2012**, *70*, 1427–1435.
109. Soga, J. The term "carcinoid" is a misnomer: the evidence based on local invasion. *J Exp Clin Cancer Res* **2009**, *28*, 15, doi:10.1186/1756-9966-28-15.

110. Tuft, L.; Girsh, L.S. Cardiac asthma--a misnomer. *J Allergy* **1960**, *31*, 519–530, doi:10.1016/0021-8707(60)90087-3.
111. Venerando, A.; Ruzzene, M.; Pinna, L.A. Casein kinase: the triple meaning of a misnomer. *Biochem J* **2014**, *460*, 141–156, doi:10.1042/bj20140178.
112. Molinaro, F.; Cerchia, E.; Bulotta, A.L.; Angotti, R.; Di Maggio, G.; Bianchi, A. Caudal 'duplication' or 'split' syndrome: Is there a misnomer? *J. Pediatr. Surg. Case Rep.* **2013**, *1*, 351–356.
113. Thakur, J.D.; Sonig, A.; Khan, I.S.; Connor, D.E., Jr.; Pait, T.G.; Nanda, A. Jacques Bénigne Winslow (1669-1760) and the misnomer cavernous sinus. *World Neurosurg* **2014**, *81*, 191–197, doi:10.1016/j.wneu.2012.06.030.
114. Cheong, W.Y.; Tan, K.P. Cerebral venous angioma--a misnomer? *Ann Acad Med Singap* **1993**, *22*, 736–741.
115. Raju, T.N. The birth of Caesar and the cesarean misnomer. *Am J Perinatol* **2007**, *24*, 567–568, doi:10.1055/s-2007-986693.
116. Sauvageau, A. The choking game: a misnomer. *Pediatr Emerg Care* **2010**, *26*, 965, doi:10.1097/PEC.0b013e3181fe923b.
117. Álvaro González, L.C. 'Chronic migraine' is a misnomer. *Neurologia* **2017**, *32*, 266–267, doi:10.1016/j.nrl.2015.06.001.
118. Kassur, B.; Januszkiewicz, J. Chronic trichinosis--a misnomer. *Przegl Epidemiol* **1970**, *24*, 153–159.
119. Fulton, C.R.; Clark, C.; Dickinson, S. Clinical Hours in Nurse Practitioner Programs Equals Clinical Competence: Fact or Mis-nomer? *Nurse Educ* **2017**, *42*, 195–198, doi:10.1097/NNE.0000000000000355.
120. "Clinical" pharmacy a misnomer. *J Indiana State Med Assoc* **1970**, *63*, 255–256.
121. Saucier, J. Concussion: a misnomer. *Can Med Assoc J* **1955**, *72*, 816–820.
122. Warnes, C.A. Congenitally corrected transposition: the uncorrected misnomer. *J Am Coll Cardiol* **1996**, *27*, 1244–1245, doi:10.1016/0735-1097(96)00037-x.
123. King, N.B. Contagion: a misnomer for financial crisis. *J Public Health (Oxf)* **2014**, *36*, 18–19, doi:10.1093/pubmed/fdt084.
124. Madden, J.L. CONVERSION OF THE PORTAL VEIN TO AN "OUTFLOW TRACT" IS A MISNOMER. *Surg Gynecol Obstet* **1963**, *117*, 499–500.
125. Roberts, W.C. Coronary "lesion," coronary "disease," "single-vessel disease," "two-vessel disease": word and phrase misnomers providing false impressions of the extent of coronary atherosclerosis in symptomatic myocardial ischemia. *Am J Cardiol* **1990**, *66*, 121–123, doi:10.1016/0002-9149(90)90751-l.
126. Moied, A.S.; Pal, J. Cricoid pressure - A misnomer in pediatric anaesthesia. *J Emerg Trauma Shock* **2010**, *3*, 96–97, doi:10.4103/0974-2700.58650.
127. Harmer, M. Crohn's disease--a misnomer? *Bristol Med Chir J* **1988**, *103*, 9–10.
128. Turner, P.J.; Holdsworth, G. CT stroke window settings: an unfortunate misleading misnomer? *Br. J. Radiol.* **2011**, *84*, 1061–1066, doi:10.1259/bjr/99730184.
129. Clement, J.G.; Blackwell, S.A. Is current bite mark analysis a misnomer? *Forensic Sci Int* **2010**, *201*, 33–37, doi:10.1016/j.forsciint.2010.03.006.
130. Wolfsohn, B.L. 'Dead Tooth' is a misnomer. *N Y J Dent* **1974**, *44*, 83.
131. Johnson, E.W. Physiatric misnomers. *Am J Phys Med Rehabil* **2001**, *80*, 161, doi:10.1097/00002060-200103000-00001.
132. Jones, M.W.; Branigan, H.P.; Hatzidaki, A.; Obregón, M. Is the 'naming' deficit in dyslexia a misnomer? *Cognition* **2010**, *116*, 56–70, doi:10.1016/j.cognition.2010.03.015.
133. Radley, J.M.; Haller, C.J. The demarcation membrane system of the megakaryocyte: a misnomer? *Blood* **1982**, *60*, 213–219.
134. Loeb, C. Dementia due to lacunar infarctions: a misnomer or a clinical entity? *Eur Neurol* **1995**, *35*, 187–192, doi:10.1159/000117126.
135. Bracha, H.S.; Vega, E.M.; Vega, C.B. Posttraumatic dental-care anxiety (PTDA): Is "dental phobia" a misnomer? *Hawaii Dent J* **2006**, *37*, 17–19.
136. Elson, S.H.; Clark, W.S.; Williams, B.R. Is 'diaphragmatic' attenuation a misnomer?. Evaluation of the anatomic cause of 'diaphragmatic' attenuation in SPECT thallium scanning. *Int J Card Imaging* **1997**, *13*, 161–164, doi:10.1023/a:1005712922362.
137. Heusch, G. Diastolic heart failure: a misNOmer. *Basic Res Cardiol* **2009**, *104*, 465–467, doi:10.1007/s00395-009-0025-3.
138. Mittelman, D. Divergence insufficiency esotropia is a misnomer. *JAMA Ophthalmol* **2013**, *131*, 547, doi:10.1001/jamaophthalmol.2013.1584.
139. Chaudhuri, Z.; Demer, J.L. Divergence insufficiency esotropia is a misnomer-reply. *JAMA Ophthalmol* **2013**, *131*, 547–548, doi:10.1001/jamaophthalmol.2013.2412.

140. Baer, P.N.; Iacono, V.J. The "DMDx test for periodontitis:" a misnomer. *J Pedod* **1988**, *13*, 68–69.
141. Rao, G. Letter: Dyskinesia of left ventricle is a misnomer. *Am J Cardiol* **1975**, *36*, 978, doi:10.1016/0002-9149(75)90095-8.
142. Koss, L.G. Dysplasia. A real concept or a misnomer? *Obstet Gynecol* **1978**, *51*, 374–379.
143. Spodick, D.H. Early repolarization: an underinvestigated misnoner. *Clin Cardiol* **1997**, *20*, 913–914, doi:10.1002/clc.4960201103.
144. Okasha, A.; Okasha, T. A plea to change the misnomer ECT. *World Psychiatry* **2014**, *13*, 327, doi:10.1002/wps.20143.
145. Magnus, R.V. Ectopia cloacae--a misnomer. *J Pediatr Surg* **1969**, *4*, 511–519, doi:10.1016/0022-3468(69)90090-6.
146. Odell, W.D. Ectopic ACTH secretion. A misnomer. *Endocrinol Metab Clin North Am* **1991**, *20*, 371–379.
147. Stammers, T. 'Elective' ventilation: an unethical and harmful misnomer? *New Bioeth* **2013**, *19*, 130–140.
148. Miller, E.C.; Kelly, M. Electric shock therapy--a misnomer. *Dis Nerv Syst* **1961**, *22*, 18–23.
149. Kakudo, K.; Bai, Y.; Liu, Z.; Ozaki, T. Encapsulated papillary thyroid carcinoma, follicular variant: a misnomer. *Pathol Int* **2012**, *62*, 155–160, doi:10.1111/j.1440-1827.2011.02773.x.
150. Vale, J.A.; Patel, A.; Ball, A.J.; Hendry, W.F.; Chappell, M.E.; Fisher, C. Endometrioid carcinoma of the prostate: a misnomer? *J R Soc Med* **1992**, *85*, 394–396.
151. Siegel, J.H. ENDOSCOPIC PAPILLOTOMY - MISNOMER. *Gastrointestinal Endoscopy* **1978**, *24*, 211.
152. Rajfer, J. Endothelial dysfunction as a cause of erectile dysfunction--misdiagnosis or misnomer? *Urology* **2004**, *64*, 193–194, doi:10.1016/j.urology.2004.04.048.
153. Sabiston, W.R. ENT: a misnomer. *Trans Sect Otolaryngol Am Acad Ophthalmol Otolaryngol* **1976**, *82*, Orl87–90.
154. Talley, N.J. The "epigastric distress syndrome:" a misnomer? *J Clin Gastroenterol* **1987**, *9*, 718, doi:10.1097/00004836-198712000-00022.
155. Endersby, R.; Albrecht, E.; Perlas, A.; Chan, V. Semantics, misnomer, or uncertainty: where is the epineurium on ultrasound? *Reg Anesth Pain Med* **2012**, *37*, 360–361, doi:10.1097/AAP.0b013e318253b48f.
156. Smith, E.T.; Pevey, J.K.; Shindler, T.O. The erector spinae transplant--a misnomer. *Clin Orthop Relat Res* **1963**, *30*, 144–151.
157. Chopra, I.J. Clinical review 86: Euthyroid sick syndrome: is it a misnomer? *J Clin Endocrinol Metab* **1997**, *82*, 329–334, doi:10.1210/jcem.82.2.3745.
158. Nogueira, G.J.; Zaglul, H.F. Hypodense extracerebral images on computed tomography in children. "External hydrocephalus": a misnomer? *Childs Nerv Syst* **1991**, *7*, 336–341, doi:10.1007/bf00304833.
159. Pandey, J.P. Fab-arm exchange is a misnomer. *MAbs* **2012**, *4*, 635, doi:10.4161/mabs.21613.
160. Johnson, E.W. The failed back syndrome (a misnomer?). *Am J Phys Med Rehabil* **1991**, *70*, 117, doi:10.1097/00002060-199106000-00001.
161. Nagy, G.K. False negative rate. A misnomer, misunderstood and misused. *Acta Cytol* **1997**, *41*, 778–780, doi:10.1159/000332703.
162. Erikssen, J.; Myhre, E. False positive exercise ECG: a misnomer? *Int J Cardiol* **1984**, *6*, 263–268, doi:10.1016/0167-5273(84)90365-6.
163. Joondeph, B.C.; Goldberg, M.F. Familial iris melanosis--a misnomer? *Br J Ophthalmol* **1989**, *73*, 289–293, doi:10.1136/bjo.73.4.289.
164. Saatci, U.; Ozen, S.; Bakkaloglu, A.; Besbas, N. Familial Mediterranean fever: a misnomer? *Lancet* **1994**, *343*, 485, doi:10.1016/s0140-6736(94)92735-9.
165. Maher, O.M.; Moonat, H.R. Fanconi Anemia and Fanconi Syndrome: Time to Correct the Misnomers. *J Pediatr Hematol Oncol* **2016**, *38*, 585, doi:10.1097/mph.0000000000000673.
166. Derbyshire, S.W.; Furedi, A. Do fetuses feel pain? "Fetal pain" is a misnomer. *Bmj* **1996**, *313*, 795, doi:10.1136/bmj.313.7060.795a.
167. Bennett, R.M. Fibrositis: misnomer for a common rheumatic disorder. *West J Med* **1981**, *134*, 405–413.
168. Food safety and the hygiene misnomer. *Perspect Public Health* **2016**, *136*, 197–198, doi:10.1177/1757913916648526.
169. Sarwar, M. Foramen of Monro: a misnomer. *AJR Am J Roentgenol* **1977**, *128*, 1069, doi:10.2214/ajr.128.6.1069.
170. Pappu, S.V. Fourier transform lens: a misnomer. *Appl Opt* **1974**, *13*, 2747, doi:10.1364/ao.13.2747_1.
171. Casey, R.G.; Galvin, D.; Bouchier-Hayes, D.; Lennon, G. Fractured penis: a clinical misnomer! *Ir J Med Sci* **2005**, *174*, 55–57, doi:10.1007/bf03168520.
172. Simson, M.B.; Spear, J.; Moore, E.N. The relationship between atrioventricular nodal refractoriness and the functional refractory period in the dog. *Circ Res* **1979**, *44*, 121–126, doi:10.1161/01.res.44.1.121.
173. Dutta, J.K. Diagnosis of gastroenteritis is a misnomer. *J Assoc Physicians India* **1994**, *42*, 847.

174. Desvaux, M.; Parham, N.J.; Scott-Tucker, A.; Henderson, I.R. The general secretory pathway: a general misnomer? *Trends Microbiol* **2004**, *12*, 306–309, doi:10.1016/j.tim.2004.05.002.
175. Olde Rikkert, M.G.; Rigaud, A.S.; van Hoeyweghen, R.J.; de Graaf, J. Geriatric syndromes: medical misnomer or progress in geriatrics? *Neth J Med* **2003**, *61*, 83–87.
176. Beckmann, J. Germ-line gene therapy a misnomer? *Nature* **1984**, *312*, 408, doi:10.1038/312408a0.
177. Duke, K.L. IS THE GERMINAL EPITHELIUM OF THE OVARY A MISNOMER. *Anat. Rec.* **1955**, *121*, 430.
178. Millard, D.W. Giggle Micturition—A Misnomer? *Br Med J* **1965**, *1*, 1674.
179. VON LACKUM, W.H.; PALOMEQUE, E.J. GONORRHEAL SPURS A MISNOMER. *JAMA* **1930**, *95*, 472–473, doi:10.1001/jama.1930.02720070010005.
180. Fisher, E.R.; Wechsler, H. Granular cell myoblastoma--a misnomer. Electron microscopic and histochemical evidence concerning its Schwann cell derivation and nature (granular cell schwannoma). *Cancer* **1962**, *15*, 936–954, doi:10.1002/1097-0142(196209/10)15:5<936::aid-cncr2820150509>3.0.co;2-f.
181. Gorchein, A.; Danton, M.; Lim, C.K. Harderoporphyrin: a misnomer. *Biomed Chromatogr* **2005**, *19*, 565–569, doi:10.1002/bmc.480.
182. Thomas, J.R. "The healthy tan": a modern misnomer. *Mo Med* **1989**, *86*, 327–328.
183. White, J.J.; Suzuki, H. Hernia through the foramen of Bochdalek: a misnomer. *J Pediatr Surg* **1972**, *7*, 60–61, doi:10.1016/0022-3468(72)90405-8.
184. Aslanger, E.; Sezer, M.; Umman, S. High blood pressure: An obscuring misnomer? *Anatol J Cardiol* **2016**, *16*, 713–719, doi:10.14744/AnatolJCardiol.2016.7054.
185. Philpot, R. Highly active antiretroviral therapy: a misnomer in HIV/AIDS terminology. *Intern Med J* **2004**, *34*, 139, doi:10.1111/j.1444-0903.2004.00557.x.
186. Bhutta, M.F.; Worley, G.A.; Harries, M.L. "Hot potato voice" in peritonsillitis: a misnomer. *J Voice* **2006**, *20*, 616–622, doi:10.1016/j.jvoice.2005.07.005.
187. Franchini, G. HTLV-1 and HIV-1 "accessory" proteins: a misleading misnomer. *Mol Aspects Med* **2010**, *31*, 331–332, doi:10.1016/j.mam.2010.08.001.
188. Buerkel, D.M.; Gurm, H.S. The human bovine arch--a common misnomer. *Catheter Cardiovasc Interv* **2007**, *70*, 162, doi:10.1002/ccd.21086.
189. Layton, K.F.; Kallmes, D.F.; Cloft, H.J.; Lindell, E.P.; Cox, V.S. Bovine aortic arch variant in humans: clarification of a common misnomer. *AJNR Am J Neuroradiol* **2006**, *27*, 1541–1542.
190. Tannenberg, J. THE HYALINE MEMBRANE SYNDROME OR DISEASE - A DANGEROUS MISNOMER. *Am. J. Pathol.* **1958**, *34*, 604–605.
191. Sambade, C.; Franssila, K.; Cameselle-Teijeiro, J.; Nesland, J.; Sobrinho-Simões, M. Hyalinizing trabecular adenoma: A misnomer for a peculiar tumor of the thyroid gland. *Endocr Pathol* **1991**, *2*, 83–91, doi:10.1007/bf02915330.
192. Parker, W. The "hygiene hypothesis" for allergic disease is a misnomer. *Bmj* **2014**, *348*, g5267, doi:10.1136/bmj.g5267.
193. Story, D.A. Hyperchloraemic acidosis: another misnomer? *Crit Care Resusc* **2004**, *6*, 188–192.
194. Opitz, J.M.; Carey, J.C. Why is the construction: Hypoplastic left heart "syndrome" a misnomer? And: What is a syndrome, anyhow? *Am J Med Genet A* **2011**, *155a*, 360–362, doi:10.1002/ajmg.a.33836.
195. Miller, L.; Ferreira, J.A.; Tucker, C. Idarucizumab for Reversal of Dabigatran-Associated Bleeding: Misnomer or Miracle? *J Emerg Med* **2017**, *52*, 341–347, doi:10.1016/j.jemermed.2016.08.023.
196. Witzleben, C.L. Idiopathic infantile arterial calcification--a misnomer? *Am J Cardiol* **1970**, *26*, 305–309, doi:10.1016/0002-9149(70)90798-8.
197. Tremolizzo, L.; Giaccone, G.; Tagliavini, F.; Ferrarese, C.; Appollonio, I. Idiopathic progressive chorea: misnomer or still reality? A case with neuropathological disconfirmation. *Neurol Sci* **2014**, *35*, 1155–1156, doi:10.1007/s10072-014-1715-7.
198. Victoriano, N.; Salvador, P. Is the so-called "immune" reaction to smallpox vaccination a misnomer? *J Philipp Med Assoc* **1952**, *28*, 348–351.
199. Cyders, M.A. The misnomer of impulsivity: commentary on "choice impulsivity" and "rapid-response impulsivity" articles by Hamilton and colleagues. *Personal Disord* **2015**, *6*, 204–205, doi:10.1037/per0000123.
200. Moore, E.N.; Boineau, J.P.; Patterson, D.F. Incomplete right bundle-branch block. An electrocardiographic enigma and possible misnomer. *Circulation* **1971**, *44*, 678–687, doi:10.1161/01.cir.44.4.678.
201. Altchek, E.D. Instep flap: misnomer? *Plast Reconstr Surg* **1984**, *73*, 501, doi:10.1097/00006534-198403000-00040.
202. Song, K.B.; Cho, S.J.; Minn, Y.K.; Kwon, K.H.; Park, M.K. A case of insulin neuritis that developed in a patient under regular insulin treatment on increasing the insulin dose. Insulin neuritis: is it a misnomer? *J Neurol* **2009**, *256*, 274–275, doi:10.1007/s00415-009-0952-9.

203. Osuntokun, B.O.; Ladipo, G.; Francis, T.I. Insulin resistance in diabetes mellitus in the African: a misnomer. *West Afr Med J Niger Pract* **1972**, *21*, 51–54.
204. McGuire, B.E.; Basten, C.J.; Ryan, C.J.; Gallagher, J. Intensive care unit syndrome: a dangerous misnomer. *Arch Intern Med* **2000**, *160*, 906–909, doi:10.1001/archinte.160.7.906.
205. Black, M.E. International health is a misnomer. *Bmj* **2013**, *347*, f4167, doi:10.1136/bmj.f4167.
206. Chew, K.K. International index of erectile function: a misnomer at risk of being misleading from misuse. *J Sex Med* **2012**, *9*, 330, doi:10.1111/j.1743-6109.2011.02357.x.
207. Castellanos, A.; Brenes, J.C.; Chirinos-Medina, J.A.; del Carpio, F. Interpolated premature ventricular contractions with postponed compensatory pauses: a misnomer? *J Electrocardiol* **2006**, *39*, 377–379, doi:10.1016/j.jelectrocard.2005.12.003.
208. Siesjö, B.K.; Pontén, U. Intracellular pH--true parameter or misnomer? *Ann N Y Acad Sci* **1966**, *133*, 78–86, doi:10.1111/j.1749-6632.1966.tb50711.x.
209. Eggleston, J.C. THE INTRAVASCULAR BRONCHIOLOALVEOLAR TUMOR AND THE SCLEROSING HEMANGIOMA OF THE LUNG - MISNOMERS OF PULMONARY NEOPLASIA. *Semin. Diagn. Pathol.* **1985**, *2*, 270–280.
210. Wiesen, A.; Hartman, D.; Talarek, C.; Gelrud, A. Iron Pill Gastritis: A Misnomer? *Am. J. Gastroenterol.* **2009**, *104*, S41.
211. Frieling, T.; Schemann, M.; Pehl, C. [Irritable bowel syndrom--a misnomer?]. *Z Gastroenterol* **2011**, *49*, 577–578, doi:10.1055/s-0031-1273323.
212. Somerville, J.; Becu, L. Proceedings: 'Isolated' pulmonary valve stenosis: a possible misnomer. *Br Heart J* **1976**, *38*, 316.
213. Baltasar, A. Laparoscopic sleeve gastrectomy is a misnomer. *Surg Obes Relat Dis* **2012**, *8*, 127, doi:10.1016/j.soard.2011.07.010.
214. Choy, D.S. Percutaneous laser disc decompression (PLDD) update: focus on device and procedure advances. *J Clin Laser Med Surg* **1993**, *11*, 181–183, doi:10.1089/clm.1993.11.181.
215. Selman, S.H. "Latent" carcinoma of the prostate: a medical misnomer? *Urology* **2000**, *56*, 708–711, doi:10.1016/s0090-4295(00)00618-x.
216. Howard, M.A. Lateral aberrant thyroid; a misnomer. *West J Surg Obstet Gynecol* **1949**, *57*, 26–28.
217. van Dam, I.; Roelandt, J.; Robles de Medina, E.O. Left atrial enlargement: an electrocardiographic misnomer? An electrocardiographic-echocardiographic study. *Eur Heart J* **1986**, *7*, 115–117, doi:10.1093/oxfordjournals.eurheartj.a062032.
218. Hoch, M.; Wang, C.; Caroline, D. Localized mediastinal amyloidosis: A misnomer? *Radiol Case Rep* **2012**, *7*, 647, doi:10.2484/rcr.v7i2.647.
219. Naini, F.B. Is "long face" a misnomer? *Am J Orthod Dentofacial Orthop* **2014**, *146*, 409–410, doi:10.1016/j.ajodo.2014.07.012.
220. Sungurtekin, U.; Ozban, M.; Erbis, H.; Birsen, O. Loose seton: a misnomer of cutting seton. *Surgical Science* **2016**, *7*, 219.
221. McManus, J.; Rutledge, G. Lower nephron nephrosis: A misnomer for the crush kidney. *Am. J. Pathol.* **1949**, *25*, 771–771.
222. Futrell, N.; Millikan, C. THE MISNOMER OF LUPUS CEREBRITIS. *Ann. Neurol.* **1989**, *26*, 158–159.
223. Bauer, J. THE MALE CLIMACTERIC—A MISNOMER. *JAMA* **1944**, *126*, 914–914, doi:10.1001/jama.1944.02850490048023.
224. Lucente, F.E.; Parisier, S.C.; Som, P.M.; Arnold, L.M. Malignant external otitis: a dangerous misnomer? *Otolaryngol Head Neck Surg* **1982**, *90*, 266–269, doi:10.1177/019459988209000223.
225. Zsigmond, E.K. Malignant hyperthermia--a misnomer. *JAMA* **1980**, *243*, 513, doi:10.1001/jama.243.6.513b.
226. Anthony, W.A. Managed care: a misnomer? *Hosp Community Psychiatry* **1993**, *44*, 794–795, doi:10.1176/ps.44.8.794.
227. Null, D.M.; deLemos, R.A. Meconium aspiration syndrome: A misnomer. *Pediatr. Res.* **1980**, *14*, 607–607.
228. Wade, D.T. Medically unexplained disability--a misnomer, and an opportunity for rehabilitation. *Clin Rehabil* **2001**, *15*, 343–347, doi:10.1191/026921501678310144.
229. Bere, E.; Brug, J. Is the term 'Mediterranean diet' a misnomer? *Public Health Nutr* **2010**, *13*, 2127–2129, doi:10.1017/s1368980010000480.
230. van Hall, E.V. The menopausal misnomer. *J Psychosom Obstet Gynaecol* **1997**, *18*, 59–62, doi:10.3109/01674829709085570.
231. Koskinas, G. [The term "mentally retarded" is a misnomer!]. *Lakartidningen* **1998**, *95*, 4836.
232. Sharma, D.; Isaji, S. Mesopancreas is a misnomer: time to correct the nomenclature. *J Hepatobiliary Pancreat Sci* **2016**, *23*, 745–749, doi:10.1002/jhbp.402.

233. Cameron, A.J. Metabolically normal obesity: a misnomer? *Int J Obes (Lond)* **2012**, *36*, 164, doi:10.1038/ijo.2011.57.
234. Davis, W.G.; Hennessy, B.; Babiera, G.; Hunt, K.; Valero, V.; Buchholz, T.A.; Sneige, N.; Gilcrease, M.Z. Metaplastic sarcomatoid carcinoma of the breast with absent or minimal overt invasive carcinomatous component: a misnomer. *Am J Surg Pathol* **2005**, *29*, 1456–1463, doi:10.1097/01.pas.0000176431.96326.49.
235. DasGupta, S.; Das, R.N.; Mishra, P.K.; Chatterjee, U.; Datta, C. Metastatic ganglioneuroma: a misnomer. *Indian J Pathol Microbiol* **2014**, *57*, 445–446, doi:10.4103/0377-4929.138753.
236. Yung, D.E.; Koulaouzidis, A.; Fineron, P.; Plevris, J.N. Microscopic colitis: a misnomer for a clearly defined entity? *Endoscopy* **2015**, *47*, 754–757, doi:10.1055/s-0034-1391986.
237. Tellier, A.; Della Malva, L.C.; Cwinn, A.; Grahovac, S.; Morrish, W.; Brennan-Barnes, M. Mild head injury: a misnomer. *Brain Inj* **1999**, *13*, 463–475, doi:10.1080/026990599121386.
238. Macfarlane, D.P.; Yu, N.; Leese, G.P. Mild primary hyperparathyroidism: a misnomer? *Expert Rev Endocrinol Metab* **2011**, *6*, 747–749, doi:10.1586/eem.11.67.
239. McDowell, C.A. Points: The so-called Miller-Fisher syndrome: a misnomer. *Br Med J (Clin Res Ed)* **1982**, *285*, 744.
240. Rose, F.C. Points: The so-called Miller-Fisher syndrome: a misnomer. *Br Med J (Clin Res Ed)* **1982**, *285*, 1051.
241. Jegodzinski, S. Misnomer "Morbi-RAS". *Radiologe* **2009**, *49*, 87.
242. Raffa, R.B.; Pergolizzi, J.V., Jr. Is morphine medicine's biggest misnomer? *Ann Pharmacother* **2012**, *46*, 1122–1123, doi:10.1345/aph.1R133.
243. Ghabili, K.; Shoja, M.M.; Golzari, S.E.; Niyousha, M.R. Mustard gas keratitis: a common misnomer. *Cornea* **2013**, *32*, 382–383, doi:10.1097/ICO.0b013e318278cdcc.
244. Talley, N.J. NERVOUS DYSPEPSIA - A MISNOMER. *Aust. N. Z. J. Med.* **1984**, *14*, 915.
245. Neuroleptic malignant syndrome (NMS): a misnomer? *Br J Psychiatry* **1987**, *151*, 863–865, doi:10.1192/bjp.151.6.863b.
246. Malhotra, V.L.; Lakshmy, A.; Kapur, S.; Partho, R. Rapidly emerging novel metallo beta lactamase--New Delhi metallo beta lactamase-1 (NDM-1)--is it a misnomer. *J Commun Dis* **2010**, *42*, 227–229.
247. Armstrong, A.; Coulston, J.; Saxby, C.; Mackey, P.; Eyre-Brook, I. Nil by mouth: a misleading misnomer. *Br. J. Surg.* **2011**, *174*, 174.
248. Ambrosio, G.; Weisman, H.F.; Becker, L.C. THE NO REFLOW PHENOMENON - A MISNOMER. *Circulation* **1986**, *74*, 260.
249. Benditt, D.G.; Milstein, S.; Burchell, H.B. Nodoventricular Accessory Connections: A Misnomer or a Structural Functional Spectrum. *J. Cardiovasc. Electrophysiol.* **1990**, *1*, 231–237, doi:10.1111/j.1540-8167.1990.tb01064.x.
250. Cullen, J.F. Re: Parsa et al.: Nonarteritic anterior ischemic optic neuropathy (NAION): a misnomer. Rearranging pieces of a puzzle to reveal a nonischemic papillopathy caused by vitreous separation (Ophthalmology 2015;122:439-42). *Ophthalmology* **2015**, *122*, e76, doi:10.1016/j.ophtha.2015.04.044.
251. Hayreh, S.S. Re: Parsa et al.: Nonarteritic anterior ischemic optic neuropathy (NAION): a misnomer. Rearranging pieces of a puzzle to reveal a nonischemic papillopathy caused by vitreous separation (Ophthalmology 2015;122:439-42). *Ophthalmology* **2015**, *122*, e75–76, doi:10.1016/j.ophtha.2015.03.039.
252. Katz, M.S.; Glaser, B.M.; Katz, B. Re: Parsa et al.: Nonarteritic anterior ischemic optic neuropathy (NAION): a misnomer. Rearranging pieces of a puzzle to reveal a nonischemic papillopathy caused by vitreous separation (Ophthalmology 2015;122:439-42). *Ophthalmology* **2015**, *122*, e73–74, doi:10.1016/j.ophtha.2015.03.041.
253. Parsa, C.F.; Hoyt, W.F. Nonarteritic anterior ischemic optic neuropathy (NAION): a misnomer. Rearranging pieces of a puzzle to reveal a nonischemic papillopathy caused by vitreous separation. *Ophthalmology* **2015**, *122*, 439–442, doi:10.1016/j.ophtha.2014.11.011.
254. Starin, S. "Nonaversive" behavior management: a misnomer. *Behav Anal* **1991**, *14*, 207–209, doi:10.1007/bf03392571.
255. Boudreau, M.D. "Nondecolorized" qualifier is a misnomer for the aloe vera whole leaf extract test material. *Toxicol Sci* **2013**, *133*, 343, doi:10.1093/toxsci/kft073.
256. van der Riet, F.D. Obese (ob) gene--a misnomer? *S Afr Med J* **1996**, *86*, 1437.
257. Kao, R.T. It's 'perio-systemic link'; 'Oral-systemic link' is a misnomer. *J Calif Dent Assoc* **2010**, *38*, 242–244.
258. Solanki, C.; Bhat, D.I.; Devi, B.I. "Os" omovertebrale variants prove it to be a misnomer. *Neurol India* **2016**, *64*, 984–987, doi:10.4103/0028-3886.190264.
259. Snidvongs, K.; Earls, P.; Dalgorf, D.; Sacks, R.; Pratt, E.; Harvey, R.J. Osteitis is a misnomer: a histopathology study in primary chronic rhinosinusitis. *Int Forum Allergy Rhinol* **2014**, *4*, 390–396, doi:10.1002/alr.21291.

260. Wurtele, P. The pale epiglottitis--a misnomer or not? *J Otolaryngol* **1984**, *13*, 406–408.
261. Lambert, L.A.; Harris, A. Palliative cytoreductive surgery and hyperthermic intraperitoneal chemoperfusion: current clinical practice or misnomer? *J Gastrointest Oncol* **2016**, *7*, 112–121, doi:10.3978/j.issn.2078-6891.2015.132.
262. Horstink, M.W.; Dekker, M.C.; Montagna, P.; Bonifati, V.; van De Warrenburg, B.P. Pallidopyramidal disease: a misnomer? *Mov Disord* **2010**, *25*, 1109–1115, doi:10.1002/mds.23118.
263. Novitzky, D.; Cooper, D.K.; Wicomb, W. Hormonal therapy to the brain-dead potential organ donor: the misnomer of the "Papworth cocktail". *Transplantation* **2008**, *86*, 1479, doi:10.1097/TP.0b013e31818c6eb5.
264. Acott, C. "Paradoxical embolism" - a misnomer? *Diving Hyperb Med* **2008**, *38*, 54–55.
265. Bromage, P.R. 'Paraplegia following epidural analgesia': a misnomer. *Anaesthesia* **1976**, *31*, 947–949, doi:10.1111/j.1365-2044.1976.tb11908.x.
266. El-Sherif, N.; Jalife, J. Paroxysmal atrioventricular block: are phase 3 and phase 4 block mechanisms or misnomers? *Heart Rhythm* **2009**, *6*, 1514–1521, doi:10.1016/j.hrthm.2009.06.025.
267. Alberti-Flor, J.J. Percutaneous endoscopic duodenostomy: a misnomer? *Gastrointest Endosc* **1989**, *35*, 582, doi:10.1016/s0016-5107(89)72927-8.
268. Verstraete, M. Peripheral vasodilator drugs: a misnomer. *Drugs* **1980**, *19*, 81–83, doi:10.2165/00003495-198019020-00001.
269. Dhossche, D.; Kellner, C.H. Pervasive refusal syndrome: A misnomer for catatonia. *Asian J Psychiatr* **2015**, *18*, 113, doi:10.1016/j.ajp.2015.09.002.
270. Cyriax, J. Physical medicine--a misnomer? *Br Med J* **1972**, *3*, 353, doi:10.1136/bmj.3.5822.353-a.
271. Hassel, B. Pickwickian syndrome, a misnomer. *Hum Pathol* **1997**, *28*, 1329–1330, doi:10.1016/s0046-8177(97)90216-1.
272. Magrath, J.L. Pilonidal sinus or cyst—A misnomer. *The American Journal of Surgery* **1944**, *64*, 101–103, doi:10.1016/S0002-9610(44)90486-1.
273. Papillon, J. [Plastic and reconstructive surgery: a misnomed specialty]. *Union Med Can* **1978**, *107*, 1125–1126.
274. Acton, V. Is pleurodesis for the treatment of primary spontaneous pneumothorax a misnomer--and if it works, does it matter? *J Thorac Cardiovasc Surg* **2015**, *149*, 397–398, doi:10.1016/j.jtcvs.2014.09.025.
275. Hughes, W.T. Pneumocystis carinii vs. Pneumocystis jiroveci: another misnomer (response to Stringer et al.). *Emerg Infect Dis* **2003**, *9*, 276–277, doi:10.3201/eid0902.020602.
276. Lam, P.M.; Raine-Fenning, N. Polycystic ovarian syndrome: a misnomer for an enigmatic disease. *Ultrasound Obstet Gynecol* **2009**, *33*, 621–627, doi:10.1002/uog.6414.
277. Karoshi, M.; Okolo, S.O. Commentary: Polycystic ovarian disease (PCOD): a misnomer, looking for a new name. *Int J Fertil Womens Med* **2004**, *49*, 191–192.
278. Coomes, E.N.; Sharp, J. Polymyalgia rheumatica: a misnomer? *Lancet* **1961**, *2*, 1328–1331, doi:10.1016/s0140-6736(61)90902-3.
279. Yeh, I.; Martin, M.A.; Robetorye, R.S.; Gunn, S. "Polysomy 17" by FISH for HER2 in breast cancer is a misnomer: Results of chromosome 17 analysis by array comparative genomic hybridization. *Cancer Res.* **2009**, *69*, 3008, doi:10.1158/0008-5472.SABCS-3008.
280. Fenton, J.E.; Turner, J.; Shirazi, A.; Fagan, P.A. Post-stapedectomy reparative granuloma: a misnomer. *J Laryngol Otol* **1996**, *110*, 185–188, doi:10.1017/s0022215100133134.
281. Rulin, M.C.; Turner, J.H.; Dunworth, R.; Thompson, D.S. Post-tubal sterilization syndrome--a misnomer. *Am J Obstet Gynecol* **1985**, *151*, 13–19, doi:10.1016/0002-9378(85)90415-6.
282. Maizlin, Z.; Shewchuk, J.; Gandehari, H.; Kirby, J.; Coblentz, C.; Maizels, L. Misnomer of Posterior Reversible Encephalopathy Syndrome—Not Only Posterior and Not Always Reversible. *AJR Am. J. Roentgenol.* **2009**, *193*, 192–206, doi:10.2214/AJR.07.2024.
283. Laban, M.M. Posterior tibial nerve: a misnomer. *Arch Phys Med Rehabil* **2011**, *92*, 1522, doi:10.1016/j.apmr.2011.06.010.
284. Sauvageau, A. Postobstructive pulmonary edema following hanging: a misnomer. *Indian J Med Sci* **2009**, *63*, 51–52.
285. Bergink, V.; Boyce, P.; Munk-Olsen, T. Postpartum psychosis: a valuable misnomer. *Aust N Z J Psychiatry* **2015**, *49*, 102–103, doi:10.1177/0004867414564698.
286. Vidne, B.A.; Lajos, T.Z.; Sayek, I.; Friedman, I.; Stoklosa, J.; Shenoy, S.; Culver, G. Postperfusion lung syndrome. A misnomer? *N Y State J Med* **1976**, *76*, 684–688.
287. Srinivas, V. Potency-sparing radical surgery: a misnomer. *Br J Urol* **1994**, *74*, 395.
288. Yaksh, T.L.; Abram, S.E. Preemptive analgesia: A popular misnomer, but a clinically relevant truth? *APS Journal* **1993**, *2*, 116–121, doi:10.1016/S1058-9139(05)80129-9.

289. Soderling, B. [Prematurity, a scientific misnomer]. *Nord Med* **1957**, *58*, 1275–1277.
290. Levi Sandri, G.B. Primary biliary cirrhosis: time to replace a misnomer. *Hepatology* **2015**, *61*, 1435, doi:10.1002/hep.27355.
291. Wahl, I.; Feige, A.; Löwe, B.; Weiler-Normann, C.; Rose, M.; Lohse, A.W.; Schramm, C. Primary biliary "cirrhosis": time to replace a misnomer. *Hepatology* **2015**, *61*, 1091, doi:10.1002/hep.27263.
292. Brooks, J.E.; Jirauch, P.M. Primary reading epilepsy: a misnomer. *Arch Neurol* **1971**, *25*, 97–104, doi:10.1001/archneur.1971.00490020015001.
293. Cockshott, W.P.; Weaver, E.J. Primary tropical splenic abscess: a misnomer. *Br J Surg* **1962**, *49*, 665–669, doi:10.1002/bjs.18004921822.
294. Fisher, E.R. Pseudomucinous cystadenoma: a misnomer? Histochemical studies on pseudomucinous cystomas and cystadenocarcinomas with special reference to their apparent mucopolysaccharide secretions. *Obstet Gynecol* **1954**, *4*, 616–621.
295. Marsh, J.L.; Naidich, T.P. PTERYGOMAXILLARY DISJUNCTION - CLARIFICATION OF AN ANATOMIC MISNOMER. *Cleft Palate Journal* **1980**, *17*, 353.
296. Scudamore, J.H.; Yates, M.J. Pudendal block--a misnomer? *Lancet* **1966**, *1*, 23–24, doi:10.1016/s0140-6736(66)90008-0.
297. Miller, C.R. Pulmonary veno-occlusive disease: a misnomer? *Pediatr Radiol* **2012**, *42*, 647–652; quiz 773–774., doi:10.1007/s00247-012-2350-1.
298. Clark, D.A.; Guyitt, B.D. 4 - Pure Obsessions: Conceptual Misnomer or Clinical Anomaly? In *Obsessive-Compulsive Disorder*, Abramowitz, J.S., McKay, D., Taylor, S., Eds.; Elsevier Science Ltd: Oxford, 2007; pp. 53–75.
299. Fechner, R.E.; Cooper, P.H.; Mills, S.E. Pyogenic granuloma of the larynx and trachea. A causal and pathologic misnomer for granulation tissue. *Arch Otolaryngol* **1981**, *107*, 30–32, doi:10.1001/archotol.1981.00790370032006.
300. Glasman, P.J.; Thuraisingam, A. Rat bite fever: a misnomer? *BMJ Case Rep* **2009**, *2009*, doi:10.1136/bcr.04.2009.1795.
301. Marks, M.M. Recurrent pilonidal disease, a misnomer; its management and treatment. *J Int Coll Surg* **1957**, *27*, 226–229.
302. Evins, S.C.; Varner, W. Renal adenoma -- a misnomer. *Urology* **1979**, *13*, 85–86, doi:10.1016/0090-4295(79)90025-6.
303. Mulè, G.; Geraci, G.; Geraci, C.; Morreale, M.; Cottone, S. The renal resistive index: is it a misnomer? *Intern Emerg Med* **2015**, *10*, 889–891, doi:10.1007/s11739-015-1323-4.
304. Sobel, B.E.; Zaman, A.K. Reperfusion injury: doublethink or misnomer. *Coron Artery Dis* **2011**, *22*, 515–517, doi:10.1097/MCA.0b013e32834ac427.
305. Nash, G.; Bowen, J.A.; Langlinais, P.C. "Respirator lung": a misnomer. *Arch Pathol* **1971**, *91*, 234–240.
306. Quintão, E.C. Is reverse cholesterol transport a misnomer for suggesting its role in the prevention of atheroma formation? *Atherosclerosis* **1995**, *116*, 1–14, doi:10.1016/0021-9150(95)05531-z.
307. Stott, V.L.; Hurrell, M.A.; Anderson, T.J. Reversible posterior leukoencephalopathy syndrome: a misnomer reviewed. *Intern Med J* **2005**, *35*, 83–90, doi:10.1111/j.1445-5994.2004.00750.x.
308. Richardson, E.H. The Richardson composite operation for uterine prolapse a misnomer. *Am. J. Obstet. Gynecol.* **1943**, *45*, 1068.
309. Lange, J.F.; Komen, N.; Akkerman, G.; Nout, E.; Horstmanshoff, H.; Schlesinger, F.; Bonjer, J.; Kleinrensink, G.J. Riolan's arch: confusing, misnomer, and obsolete. A literature survey of the connection(s) between the superior and inferior mesenteric arteries. *Am J Surg* **2007**, *193*, 742–748, doi:10.1016/j.amjsurg.2006.10.022.
310. Durkin, J.F. Secondary cartilage: a misnomer? *Am J Orthod* **1972**, *62*, 15–41, doi:10.1016/0002-9416(72)90122-4.
311. Arora, S.S.; Paul, S.; Arora, S.; Kapoor, V. Secondary jaw aneurysmal bone cyst (JABC)--a possible misnomer? A review of literature on secondary JABCs, their pathogenesis and oncogenesis. *J Oral Pathol Med* **2014**, *43*, 647–651, doi:10.1111/jop.12132.
312. Berkowitz, R.L.; Lynch, L. Selective reduction: an unfortunate misnomer. *Obstet Gynecol* **1990**, *75*, 873–874.
313. Thurman, R.H.; Chervenak, F.A.; McCullough, L.B.; Halwani, S.; Farine, D. Self-plagiarism: a misnomer. *Am J Obstet Gynecol* **2016**, *214*, 91–93, doi:10.1016/j.ajog.2015.09.004.
314. Andreescu, L. Self-plagiarism in academic publishing: the anatomy of a misnomer. *Sci Eng Ethics* **2013**, *19*, 775–797, doi:10.1007/s11948-012-9416-1.
315. Nelson, R.O.; Hayes, S.C.; Spong, R.T.; Jarrett, R.B.; McKnight, D.L. Self-reinforcement: appealing misnomer or effective mechanism? *Behav Res Ther* **1983**, *21*, 557–566, doi:10.1016/0005-7967(83)90047-5.

316. Ryan, M.D.; Taylor, T.K. Septic discitis--a misnomer. *Med J Aust* **1987**, *147*, 415.
317. Duhaime, A.C.; Gennarelli, T.A.; Sutton, L.M.; Schut, L. The «shaken baby syndrome»: A misnomer? *Rivista di Neuroscienze Pediatriche* **1988**, *4*, 77–86.
318. Joyce, C.R. "Side" effects: a misnomer. *J Med Ethics* **1976**, *2*, 112–117.
319. Clarke, L.; Zelhof, B.; Fowler, S.M.; Napier-Hemy, R.D.; Burke, D.M.; McIntyre, I.G. The simple nephrectomy: A misnomer? *Eur. Urol. Suppl.* **2014**, *13*, e272.
320. Johnson, E.W.; Vandenhoven, R.P. SKELETAL-MUSCLE SPASM - A CONCEPTUAL AND PHYSIOLOGIC MISNOMER. *Arch. Phys. Med. Rehabil.* **1980**, *61*, 497.
321. Kumar, K. Should slipped capital femoral epiphysis (SCFE), a misnomer, be renamed as idiopathic capital femoral physiolysis (ICFP). *J Bone Joint Surg Br* **2002**, *84*, 932.
322. Hall, T.C.; Hacker, B.; Kessel, D. SLOWLY PROLIFERATING ACUTE LEUKEMIA - MISNOMER. *Proceedings of the American Association for Cancer Research* **1972**, *13*, 88.
323. Bucknall, R.A. "Species specificity" of interferons: a misnomer? *Nature* **1967**, *216*, 1022–1023, doi:10.1038/2161022a0.
324. Spencer, M.; Quinn, J.M.; Wagner, R.K. Specific Reading Comprehension Disability: Major Problem, Myth, or Misnomer? *Learn Disabil Res Pract* **2014**, *29*, 3–9, doi:10.1111/ldrp.12024.
325. Quintini, C.; Hirose, K.; Hashimoto, K.; Diago, T.; Aucejo, F.; Eghtesad, B.; Vogt, D.; Pierce, G.; Baker, M.; Kelly, D.; et al. "Splenic artery steal syndrome" is a misnomer: the cause is portal hyperperfusion, not arterial siphon. *Liver Transpl* **2008**, *14*, 374–379, doi:10.1002/lt.21386.
326. Bowyer, R.C.; Touquet, V.L. Spontaneous sternal fracture--a misnomer. *J R Soc Med* **1986**, *79*, 175–176, doi:10.1177/014107688607900316.
327. de Escobar, G.M. Sporadic cretinism: a dangerous misnomer. *Eur Thyroid J* **2013**, *2*, 68, doi:10.1159/000348249.
328. MacEachern, A.G.; Heyse-Moore, G.H. Stable intertrochanteric femoral fractures. A misnomer? *J Bone Joint Surg Br* **1983**, *65*, 582–583, doi:10.1302/0301-620x.65b5.6643561.
329. Sheridan, J.J. The stethoscope--Laennec's misnomer. *J R Coll Physicians Lond* **1990**, *24*, 318.
330. Spuijbroek, E.J.; Blom, N.; Braam, A.W.; Kahn, D.A. Stockholm syndrome manifestation of Munchausen: an eye-catching misnomer. *J Psychiatr Pract* **2012**, *18*, 296–303, doi:10.1097/01.pra.0000416021.68462.f3.
331. Kuhn, R.A. The misnomer "stroke". *Am Pract Dig Treat* **1960**, *11*, 270–277.
332. Duntas, L.H. Subclinical hypothyroidism: a misnomer in search of a new name. *Thyroid* **2001**, *11*, 361–362, doi:10.1089/10507250152039091.
333. Mashhadi, S.A.; Loh, C.Y.Y. Subcuticular suture—is it a misnomer? *Eur. J. Plast. Surg.* **2010**, *33*, 233–233, doi:10.1007/s00238-010-0448-y.
334. Bundens, W.P.; Bergan, J.J.; Halasz, N.A.; Murray, J.; Drehobl, M. The superficial femoral vein. A potentially lethal misnomer. *JAMA* **1995**, *274*, 1296–1298.
335. Breen, L.A.; Gutmann, L.; Riggs, J.E. Superior oblique myokymia. A misnomer. *J Clin Neuroophthalmol* **1983**, *3*, 131–132.
336. Phipps, S.; Lopez-Cordova, N.; Long, A.; Larson, S.; Lensing, S.; Rai, S.N. Symptoms of post-traumatic stress disorder in children with cancer and their parents: A misnomer? *Psychosom. Med.* **2002**, *64*, 156–157.
337. Miettinen, M.; Virtanen, I. Synovial sarcoma--a misnomer. *Am J Pathol* **1984**, *117*, 18–25.
338. Choo, H.; Kim, S.H.; Huang, J.C. TAD, a misnomer? *Am J Orthod Dentofacial Orthop* **2009**, *136*, 145–146, doi:10.1016/j.ajodo.2009.06.011.
339. Perry, E.; Ranzino, A. Tendinitis: A misnomer? ; 2011; pp. 30–36.
340. King, B.; Wollaston, J.F.; Gillanders, T.G. Tenosynovitis in industry: menace or misnomer? *Br Med J (Clin Res Ed)* **1987**, *295*, 501, doi:10.1136/bmj.295.6596.501.
341. Evans, G. Tenosynovitis in industry: menace or misnomer? *Br Med J (Clin Res Ed)* **1987**, *294*, 1569–1570, doi:10.1136/bmj.294.6587.1569-a.
342. Weinberg, A.G. "Teratomas" in the Currarino Triad: A Misnomer. *Pediatr. Dev. Pathol.* **2000**, *3*, 110–111, doi:10.1007/s100249910015.
343. Chou, H.C.; Wu, T.L. Thyromental distance and anterior larynx: misconception and misnomer? *Anesth Analg* **2003**, *96*, 1526–1527, doi:10.1213/01.Ane.0000051699.24104.85.
344. Gage, A.A. "TMJ"--often used as a misnomer. *N Y State Dent J* **1983**, *49*, 299–301.
345. Manor, D.; Atkinson, J. Is tocopherol associated protein a misnomer? *J Nutr Biochem* **2003**, *14*, 421–422, doi:10.1016/s0955-2863(03)00073-1.
346. Francis, G. Gesellschaft and the hospital: is total care a misnomer? *ANS Adv Nurs Sci* **1980**, *2*, 9–13, doi:10.1097/00012272-198007000-00003.

347. Morgado, P.J. Total mesorectal excision: a misnomer for a sound surgical approach. *Dis Colon Rectum* **1998**, *41*, 120–121, doi:10.1007/bf02236909.
348. Koss, L.G. Traditional cell metaplasia of cervix: a misnomer. *Am J Surg Pathol* **1998**, *22*, 774–776, doi:10.1097/00000478-199806000-00020.
349. Hartrick, C.T. Transient radicular irritation: a misnomer? *Anesth Analg* **1997**, *84*, 1392–1393, doi:10.1097/00000539-199706000-00050.
350. WESSON, M.B. "TRAUMATIC ORCHITIS": A MISNOMER. *JAMA* **1928**, *91*, 1857–1861, doi:10.1001/jama.1928.02700240011004.
351. Delarue, N. Traumatic unconsciousness, a clinical misnomer; present concepts of states of impaired consciousness. *Can. Med. Assoc. J.* **1965**, *93*, 545–550.
352. Fink, M. The "treatment-resistant" label in bipolar disorder is a misnomer. *Psychiatric Annals* **2005**, *35*, 965–969.
353. McCarthy, N.; Fau, M.G.; Fau, B.M.; Fau, W.N.; Lindeman, G.J. Triple-negative breast cancer: making the most of a misnomer. *Asia Pac. J. Clin. Oncol. 8*, 144–155, doi:10.1111/j.1743-7563.2012.01533.x.
354. Board, T.N.; Hughes, S.J.; Freemont, A.J. Trochanteric bursitis: the last great misnomer. *Hip Int* **2014**, *24*, 610–615, doi:10.5301/hipint.5000154.
355. Parks, G.A.; Dumars, K.W.; Limbeck, G.A.; Quinlivan, W.L.; New, M.I. "True agonadism": a misnomer? *J Pediatr* **1974**, *84*, 375–380, doi:10.1016/s0022-3476(74)80720-1.
356. Parente, J.T.; Costello, C. Tubal ligation: a misnomer. *Am J Obstet Gynecol* **1985**, *151*, 829, doi:10.1016/0002-9378(85)90536-8.
357. Parton, M.; Das, T.; Sa, G.; Finke, J.; Eisen, T.; Tannenbaum, C. Tumour necrosis factor – Misnomer and therapeutic target. In *Renal Cell Carcinoma: Molecular Targets and Clinical Applications*; Humana Press: New York, NY, USA, 2009; pp. 425–448.
358. Surawicz, B. U wave: facts, hypotheses, misconceptions, and misnomers. *J Cardiovasc Electrophysiol* **1998**, *9*, 1117–1128, doi:10.1111/j.1540-8167.1998.tb00890.x.
359. Jenson, A.B.; Fechner, R.E. Ultrastructure of an intermediate Sertoli-Leydig cell tumor. A histogenetic misnomer. *Lab. Invest. 21*, 527–535.
360. Liegl-Atzwanger, B.; Hofmann, G.; Leithner, A.; Beham, A. Undifferentiated high-grade pleomorphic sarcoma (UHPS): Diagnostic criteria, differential diagnosis, and treatment. An attempt to erasure the misnomer "MFH". *Eur. Surg.* **2009**, *41*, 143–149, doi:10.1007/s10353-009-0474-9.
361. Puschmann, A. Unverricht-Lundborg disease-a misnomer? *Mov Disord* **2009**, *24*, 629–630, doi:10.1002/mds.22119.
362. Klyce, S.D. UVA-riboflavin collagen cross-linking: a misnomer perhaps, but it works! *Invest Ophthalmol Vis Sci* **2013**, *54*, doi:10.1167/iovs.13-11807.
363. Worthington, C. Valsalva maneuver: a misnomer? *J Neurosurg* **1991**, *75*, 497, doi:10.3171/jns.1991.75.3.0497.
364. Mudry, A. Valsalva manoeuvre: A confusing dichotomatic misnomer. *Int J Pediatr Otorhinolaryngol* **2015**, *79*, 1851–1855, doi:10.1016/j.ijporl.2015.08.023.
365. Breithardt, G.; Baumgartner, H. Valvular heart disease among non-valvular atrial fibrillation: a misnomer, in search of a new term. *Eur Heart J* **2015**, *36*, 1794–1797, doi:10.1093/eurheartj/ehv193.
366. Huang, Y.P.; Chaudhary, M.Y.; Patel, S.; Yang, W. VENOUS ANGIOMA - A MISNOMER QUESTIONABLE CLINICAL AND RADIOLOGICAL FEATURES. *AJNR Am. J. Neuroradiol.* **1980**, *1*, 124–125.
367. Adelani, M.A.; Keeney, J.A.; Nunley, R.M.; Clohisy, J.C.; Barrack, R.L. Readmission following total knee arthroplasty: venous thromboembolism as a "never event" is a counterproductive misnomer. *J Arthroplasty* **2013**, *28*, 747–750, doi:10.1016/j.arth.2013.01.006.
368. Glanz, H.; Kleinsasser, O. Verrucous carcinoma of the larynx--a misnomer. *Arch Otorhinolaryngol* **1987**, *244*, 108–111, doi:10.1007/bf00458559.
369. Barer, M.R. Viable but non-culturable and dormant bacteria: time to resolve an oxymoron and a misnomer? *J Med Microbiol* **1997**, *46*, 629–631, doi:10.1099/00222615-46-8-629.
370. Spraul, C.W. [Wave front correction: a wrong promise or just a misnomer]. *Klin Monbl Augenheilkd* **2004**, *221*, 455, doi:10.1055/s-2004-812975.
371. Yarze, J.C. White bile: a "clear" misnomer. *Gastrointest Endosc* **2002**, *56*, 782–783, doi:10.1067/mge.2002.128701.
372. Mancia, G.; Zanchetti, A. White-coat hypertension: misnomers, misconceptions and misunderstandings. What should we do next? *J Hypertens* **1996**, *14*, 1049–1052, doi:10.1097/00004872-199609000-00001.

373. AbouZeid, A.A.; Mohammad, S.A.; Radwan, N.A.; Safoury, H.S.; El-Naggar, O.; Hay, S.A. Y-Type Urethral Duplication: A True Variant of the Anomaly or a Misnomer? *Eur J Pediatr Surg* **2016**, *26*, 245–251, doi:10.1055/s-0035-1551569.
